# Software comparison for evaluating genomic copy number variation for Affymetrix 6.0 SNP array platform

**DOI:** 10.1186/1471-2105-12-220

**Published:** 2011-05-31

**Authors:** Jeanette E Eckel-Passow, Elizabeth J Atkinson, Sooraj Maharjan, Sharon LR Kardia, Mariza de Andrade

**Affiliations:** 1 Division of Biomedical Statistics and Informatics, Mayo Clinic, 200 First Street SW, Rochester, MN 55905, USA; 2 Department of Epidemiology, University of Michigan, Ann Arbor, Michigan 48109, USA

## Abstract

**Background:**

Copy number data are routinely being extracted from genome-wide association study chips using a variety of software. We empirically evaluated and compared four freely-available software packages designed for Affymetrix SNP chips to estimate copy number: Affymetrix Power Tools (APT), Aroma.Affymetrix, PennCNV and CRLMM. Our evaluation used 1,418 GENOA samples that were genotyped on the Affymetrix Genome-Wide Human SNP Array 6.0. We compared bias and variance in the locus-level copy number data, the concordance amongst regions of copy number gains/deletions and the false-positive rate amongst deleted segments.

**Results:**

APT had median locus-level copy numbers closest to a value of two, whereas PennCNV and Aroma.Affymetrix had the smallest variability associated with the median copy number. Of those evaluated, only PennCNV provides copy number specific quality-control metrics and identified 136 poor CNV samples. Regions of copy number variation (CNV) were detected using the hidden Markov models provided within PennCNV and CRLMM/VanillaIce. PennCNV detected more CNVs than CRLMM/VanillaIce; the median number of CNVs detected per sample was 39 and 30, respectively. PennCNV detected most of the regions that CRLMM/VanillaIce did as well as additional CNV regions. The median concordance between PennCNV and CRLMM/VanillaIce was 47.9% for duplications and 51.5% for deletions. The estimated false-positive rate associated with deletions was similar for PennCNV and CRLMM/VanillaIce.

**Conclusions:**

If the objective is to perform statistical tests on the locus-level copy number data, our empirical results suggest that PennCNV or Aroma.Affymetrix is optimal. If the objective is to perform statistical tests on the summarized segmented data then PennCNV would be preferred over CRLMM/VanillaIce. Specifically, PennCNV allows the analyst to estimate locus-level copy number, perform segmentation and evaluate CNV-specific quality-control metrics within a single software package. PennCNV has relatively small bias, small variability and detects more regions while maintaining a similar estimated false-positive rate as CRLMM/VanillaIce. More generally, we advocate that software developers need to provide guidance with respect to evaluating and choosing optimal settings in order to obtain optimal results for an individual dataset. Until such guidance exists, we recommend trying multiple algorithms, evaluating concordance/discordance and subsequently consider the union of regions for downstream association tests.

## Background

Data from genome-wide association studies are now being mined for genotyping as well as for copy number estimation. Often, the primary objective is genotyping and secondarily the data are interrogated to evaluate copy number variation (CNV). It is well recognized that the genotyping algorithms are usually highly concordant for most SNPs, particularly for the two most common SNP array vendors: Illumina and Affymetrix. However, although the probe intensities produce concordant SNP calls, this does not imply that the probe intensities are sufficiently clean to accurately estimate copy number. In fact, copy number estimation is more susceptible than genotyping to variability in sample processing procedures as well as analytical processing steps. For example, copy number data obtained from SNP arrays are highly prone to batch effects, which reflect systematic variability during sample processing [1]. Although genotype clustering algorithms are relatively robust to these batch effects, copy number data are not. This systematic variability will result in biased estimates of copy number and thus can negatively impact downstream statistical analyses.

There are numerous software packages available for genotyping and for copy number estimation, of which most have options available for removing systematic variability. However, each software package uses different algorithms for removing this variation resulting in different effects on downstream statistical analyses. In an attempt to compare CNV software for oligonulceotide data, Baross et al. [2] compared four software packages for preprocessing and analyzing CNV data obtained from the Affymetrix 100K SNP array platform: Copy Number Analyzer for GeneChip (CNAG 1.1), dChip, Affymetrix GeneChip^® ^Chromosome Copy Number Analysis Tool (CNAT 3.0), and Gain and Loss Analysis of DNA (GLAD). Baross and colleagues found that the numbers and types of CNVs varied significantly across the four software packages and thus concluded that at least two software packages are necessary in order to identify all real copy number aberrations. Their assumption is that the algorithms have different strengths and thus taking the union of all the copy number aberrations should increase sensitivity. More recently, Winchester et al. [3] compared various freely- and commercially-available software for detecting germline CNVs using both Illumina and Affymetrix SNP arrays. With respect to freely-available software they primarily evaluated QuantiSNP and PennCNV. Winchester and colleagues also suggest applying two algorithms on a single dataset in order to obtain the most informative results and recommend using software that was designed specifically for the CNV array platform being used.

Both Baross et al. [2] and Winchester et al. [3] compared results obtained after segmentation, and thus did not evaluate the agreement, or lack thereof, in the locus-level copy number data. Furthermore, since the Baross et al. [2] publication, the technology has advanced and there have been additional algorithms developed that are now routinely used. Thus, the goal of this paper is to compare freely-available software that is routinely used for CNV data obtained from the Affymetrix Genome-Wide Human SNP Array 6.0 platform, the newest Affymetrix SNP platform that has an order of magnitude more probes than the older 100K platform. The SNP Array 6.0 has 1.8 million genetic markers, including more than 906,600 SNPs and 946,000 probes for the detection of CNV.

Herein, we provide a description of four freely-available software packages that are commonly used for CNV analysis of data generated from Affymetrix Genome-Wide Human SNP Array 6.0 platform. We compare both the bias and variance in the locus-level copy number data as well as concordance of the segmentation results obtained using a hidden Markov model (HMM). Throughout, we report on results from autosomal chromosomes only and we often use chromosome 22 as an informative example.

## Results and Discussion

We used a germline dataset consisting of 1,418 Caucasian samples to empirically evaluate four software packages developed for the Affymetrix 6.0 SNP array platform. The four software packages evaluated were PennCNV [4], Aroma.Affymetrix [5], Affymetrix Power Tools (APT) [6] and Corrected Robust Linear Model with Maximum Likelihood Distance (CRLMM) [1]. It is important to note that PennCNV, Aroma.Affymetrix and APT estimate relative copy number, relative to a reference sample or cohort of reference samples. In contrast, CRLMM estimates absolute copy number. Scharpf et al. [1] advocates estimating absolute copy number in comparison to relative copy number stating that the disadvantages of estimating relative copy number are that a reference set is necessary, a deviation from the normal two copies can either represent an aberration in the test sample or the reference set, and lastly, that the allelic copy number at polymorphic loci is often ignored.

As discussed above, copy number estimation is very susceptible to variability in the analytical processing steps. Analytical processing can entail numerous steps including, but not limited to, background correction, normalization, genomic wave correction, batch effect removal and choice of denominator in calculating relative copy number. A list of the default settings for each of the analytical processing steps for the four software packages is presented in Table 1 and discussed in detail below.

**Table 1:** List of CNV analysis software

Software	CN	**locus-level CN (LRR = Log**_**2**_**R)**^†^	Normalization (default target distribution)	Genomic-wave or GC correction	Batch-effect removal
PennCNV	Relative	*R = (A + B)/ R_exp_	Quantile (HapMap)	-	-
Aroma.Affymetrix	Relative	R = (A + B)/ median(A + B)	First, calibrates for offset and allelic crosstalk. Second, performs quantile (self).	Post normalization, corrects for PCR fragment length and GC content.	-
Affymetrix Power Tools (APT)	Relative	R = (A + B)/ median(A + B)	Quantile (self)	-	-
CRLMM	Absolute	**Linear model	Quantile (HapMap)	-	Standard argument to specify in the linear model

^† ^A denotes the A-allele intensity and B the B-allele intensity for the corresponding probe.

* R_exp _is computed from linear interpolation of canonical genotype clusters [4].

** Corrects for optical noise and non-specific binding in the linear model.

***Log***_***2***_***R ***denotes the relative locus-level copy number of the sample of interest relative to a reference sample(s). PennCNV utilizes the HapMap samples as the reference data (denominator) for calculating Log_2_R, whereas Aroma.Affymetrix and APT utilize the data at hand as their reference data. CRLMM estimates absolute copy number using a linear model and thus does not require a reference sample(s); according to their documentation, CRLMM requires at least 10 samples in order to obtain accurate estimates of the model parameters.

***Background correction ***attempts to remove optical background and non-specific hybridization [7]. PennCNV and APT do not correct for background hybridization. Aroma.Affymetrix corrects for allelic crosstalk prior to performing quantile normalization and CRLMM corrects for optical background and non-specific hybridization using a linear model after performing quantile normalization.

***Across-array normalization ***attempts to correct for systematic variability induced by array manufacturing, sample preparation, and labelling, hybridization and scanning of the arrays [7]. By default, all four software packages apply quantile normalization, which makes the distribution of probe intensities for each array equivalent [8]. Although each of the software applies quantile normalization, they utilize different target distributions. Particularly, Aroma.Affymetrix and APT utilize the data at hand to define the target distribution, whereas PennCNV and CRLMM use the HapMap samples to define a target distribution.

***Genomic-wave ***is an artefact that has been observed in both array comparative genomic hybridization (aCGH) and SNP data and is thought to be correlated with GC content [9,10]. By default, only Aroma.Affymetrix corrects for genomic waves; however, options are available in PennCNV and APT to correct for genomic waves. Specifically, Aroma.Affymetrix corrects for GC content and PCR fragment length to the post quantile-normalized data.

***Batch effect*s **are an artefact of processing samples in multiple laboratories, by multiple technicians, using reagents from multiple batches, or other sample processing steps that are not constant across samples and effects individual probes differently [1]. Quantile normalization corrects for global systematic effects, whereas the goal of batch-effect removal is to correct for probe-specific artefacts. Of the four software evaluated, only CRLMM has an option to correct for batch effects; Scharpf and colleagues assume that batch can be easily identified and thus model batch as a fixed effect [1]. For the empirical data used herein, batch was defined according to the 96-well plate that the sample was processed on. As a preliminary evaluation, for each locus on chromosome 22, we performed analysis of variance (ANOVA) on the post-processed locus-level copy number data by 96-well plate; there were 23 plates utilized in the GENOA study. The post-processed locus-level copy number data produced by PennCNV and CRLMM resulted in larger F-statistics in comparison to the post-processed data from Aroma.Affymetrix and APT (Table 2). Furthermore, although CRLMM corrects for batch effects as part of the analytical processing, locus-level batch effects are still present in the post-processed data.

**Table 2:** Summary of all F-statistics for chromosome 22 from performing analysis of variance (ANOVA) on the locus-level copy number data by 96-well plate; there were 23 plates utilized in the GENOA study

	Quantile				
**Software**	**0% (Min)**	**25%**	**50% (Median)**	**75%**	**100% (Max)**

PennCNV	0.866	21.600	32.580	47.830	224.000

Aroma.Affymetrix	0.547	2.558	2.590	4.929	19.120

APT	0.463	2.614	3.650	5.052	19.040

CLRMM	1.165	19.840	29.830	44.390	203.400

The ANOVA provides an F-statistic with 22 and 1417 degrees of freedom for each locus; the associated critical value for the 0.999 quantile is 2.21595.

**Figure 1:**
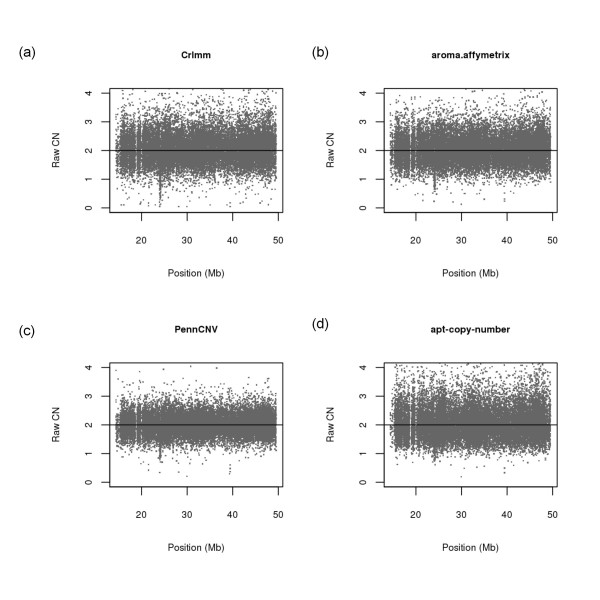
**Chromosome 22 locus-level copy number**. Data from a single representative sample are displayed using (a) CRLMM (b) Aroma.Affymetrix (c) PennCNV and (d) Affy Power Tools. The sample was randomly chosen from the available 1,418 samples.

An example of the post-processed locus-level copy number data for chromosome 22 is displayed in Figure 1 for each of the four software packages. Each point in Figure 1 represents a locus (SNP) on the Affymetrix 6.0 SNP array. As expected for germline data, the locus-level copy number data are randomly scattered around a value of two, which represents the normal two-copy state.

### Comparison of bias and variance of locus-level data

**Figure 2:**
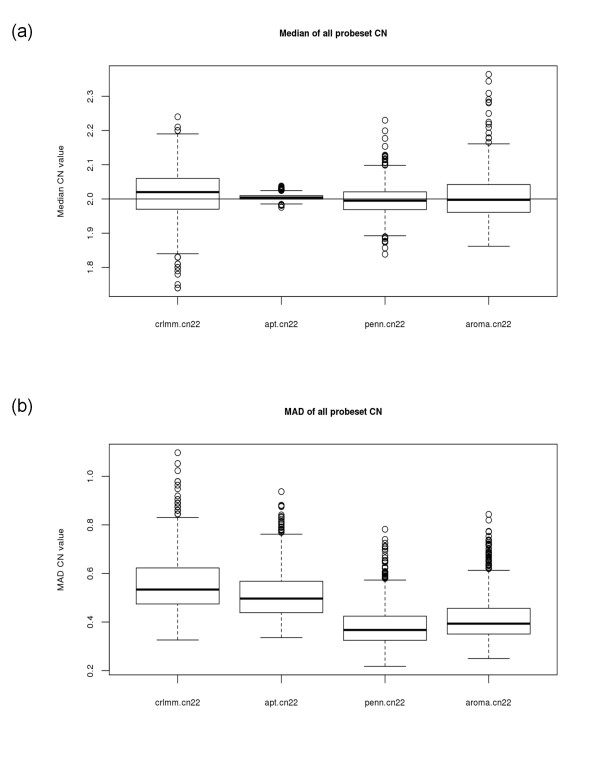
**Median locus-level copy number and median absolute deviation (MAD) for chromosome 22 across all 1,418 samples for each of the four software packages**. Results from all samples for the four packages using (a) median locus-level copy number and (b) MAD

The distribution, across the 1,418 samples, of the median locus-level copy number (both polymorphic and non-polymorphic) for chromosome 22 is presented in Figure 2a. On average, CRLMM produced slightly larger median locus-level copy number (median = 2.02) in comparison to APT (median = 2.004), PennCNV (median = 1.995) and Aroma.Affymetrix (median = 1.998). Across the 1,418 samples, APT most consistently obtained a median copy number of two, ranging from 1.976 to 2.043. Of note, samples with a median copy number larger than 2.1 or less than 1.9 were not from the same 96-well plate nor did the same samples consistently produce large (small) median copy numbers across all four software. Figure 2b provides the distribution of the median absolute deviation (MAD) on chromosome 22 for each of the 1,418 samples. The MAD is a robust measure of variability and is defined as the median of the absolute deviations from the data's median. Thus, the MAD is the variability about the median as the standard deviation is the variability about the mean. The locus-level copy number data produced by PennCNV and Aroma.Affymetrix are less variable, on average, (median = 0.37 and 0.39 respectively) in comparison to CRLMM and APT (median = 0.53 and 0.50 respectively). The derivative log ratio spread (DLRS), another robust estimator, was also used to evaluate variability and produced results similar to MAD (data not shown). To summarize, although APT produced median locus-level copy numbers closest to the normal two-copy state across all samples evaluated (Figure 2a), the locus-level copy numbers produced by APT are more variable in comparison to PennCNV and Aroma.Affymetrix (Figure 2b). The results are similar across all autosomes (data not shown).

### Agreement of locus-level copy number data

**Figure 3:**
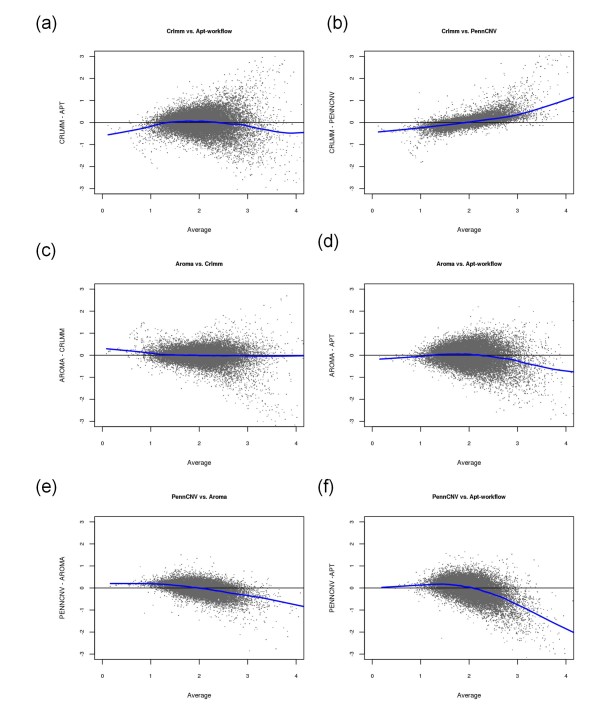
**Bland-Altman plots comparing the four software packages for a single representative sample**. Data from chromosome 22 for a single representative sample are displayed comparing (a) CRLMM and APT, (b) CRLMM and PennCNV, (c) Aroma.Affymetrix and CRLMM, (d) Aroma.Affymetrix and APT, (e) PennCNV and Aroma.Affymetrix, and (f) PennCNV and APT. The points represent locus-level copy number and the line denotes a locally-weighted average to the points.

Bland-Altman plots [11] comparing the four software packages for a single representative sample for chromosome 22 are presented in Figure 3. Each point on the plot denotes a locus on chromosome 22 and the line represents a locally-weighted average (referred to hereafter as a loess line). If the differences in estimated copy number between two software packages are not related to the magnitude of either copy number measurement, then it is expected that the data will be randomly scattered around the zero horizontal reference line. For the representative sample displayed in Figure 3, the difference in the locus-level copy number between any two software packages is in fact related to the magnitude of copy number; with the exception of the comparison of Aroma.Affymetrix and CRLMM (Figure 3c) Particularly, the software packages tend to disagree most for copy number values larger than the normal two-copy state. The exception being Aroma.Affymetrix and CRLMM, where the average differences between the locus-level copy number is approximately zero, as suggested by the loess line that nearly perfectly overlays the zero horizontal reference line.

**Figure 4:**
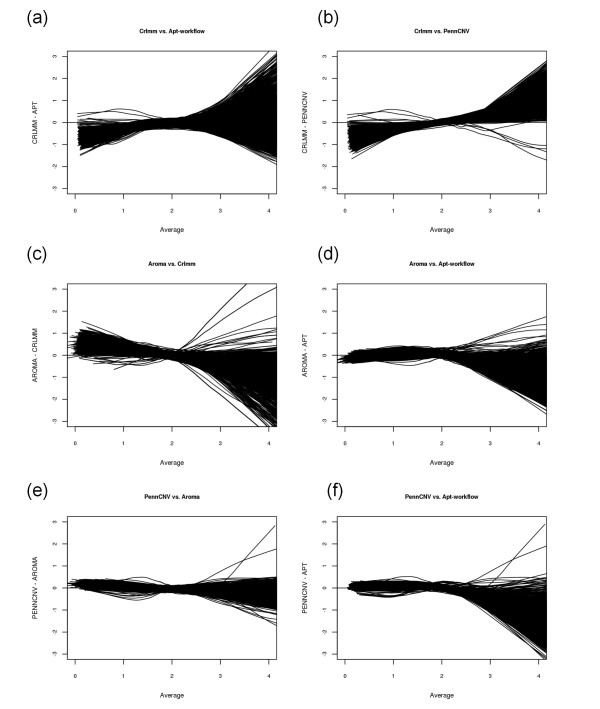
**Bland-Altman plots comparing the four software packages across all 1,418 samples**. Data from chromosome 22 comparing (a) CRLMM and APT, (b) CRLMM and PennCNV, (c) Aroma.Affymetrix and CRLMM, (d) Aroma.Affymetrix and APT, (e) PennCNV and Aroma.Affymetrix, and (f) PennCNV and APT. Each line denotes a sample.

Whereas Figure 3 displays data for a single sample, Figure 4 displays Bland-Altman plots across all 1,418 samples for chromosome 22. The individual locus-level data points are not plotted in Figure 4; instead, a loess line is provided for each sample. In general, all samples tend to follow a similar trend. Particularly, the software packages tend to disagree most for copy number values less than one and copy number values larger than three. Furthermore, although Aroma.Affymetrix and CRLMM appeared to produce the most similar locus-level copy number values on average for the single representative sample displayed in Figure 3c, this was not universally true across all 1,418 samples studied (Figure 4c). For the data studied here, PennCNV and Aroma.Affymetrix produced the most similar locus-level copy number values on average (Figure 4e).

Up to this point, for ease of explanation, we have only used data from chromosome 22 to compare the four software programs. Similar trends were observed across the other autosomes (data not shown).

### Concordance of detected segmentation regions

In addition to evaluating the agreement of locus-level copy number data across the different software, we also evaluated the concordance (or discordance) of identified regions of copy number gain and loss as obtained from the HMM algorithm. Here, we only compare the results obtained from CRLMM and PennCNV; of the four software packages evaluated, they are the only two that contain a segmentation algorithm. Although CRLMM itself does not contain a segmentation algorithm, the same authors developed VanillaIce [12] and thus the results from CRLMM can be directly imported into VanillaIce without extra effort (hereafter referred to as CRLMM/VanillaIce).

It is important to note that while neither CRLMM/VanillaIce nor PennCNV apply CNV-specific quality-control metrics to the segmented data by default, only PennCNV provides CNV-specific quality-control metrics that can be used to identify potentially poor CNV samples (see Methods). Of the 1,418 samples analyzed, 136 samples were identified as potentially poor CNV samples in the PennCNV analysis. CRLMM suggests using a signal-to-noise measure to identify samples that have poor quality for genotype calling but do not have additional CNV-specific quality-control measures. Using the signal-to-noise measure, CRLMM identified 51 samples that were poor samples for genotyping purposes, 35 of which PennCNV also identified as poor samples. The 152 samples identified as poor samples via PennCNV and CRLMM were removed when evaluating concordance between PennCNV and CRLMM/VanillaIce.

A summary of the number of CNVs that were detected for CRLMM/VanillaIce and PennCNV across the 1,266 samples that passed the PennCNV CNV-specific quality-control metrics is presented in Table 3. Overall, PennCNV detected more CNVs than CRLMM/VanillaIce; the median number of CNVs detected per sample was 39 and 30, respectively. Furthermore, the maximum number of CNVs per patient was 96 by PennCNV and 438 by CRLMM/VanillaIce. Remember, a sample was eliminated if it had more than 100 CNVs detected by PennCNV. Thus, there is at least one sample in which CRLMM/VanillaIce detected 438 CNVs but PennCNV detected less than or equal to 100 CNVs. Lastly, both packages detected many more deletions than amplifications, which others have also observed [13,14]. The median number of loci (SNPs) contained within a segment for CRLMM/VanillaIce was 15 (ranged from 1 to 6559 probes), whereas the median number of loci contained within a PennCNV segment was 32 (ranged from 3 to 6352).

**Table 3:** Summary of CNV regions on chromosome 22 using HMM algorithm for 1,266 subjects that passed PennCNV and CRLMM QC metrics

Software	Number CNVs	Duplications	Deletions	Median Number CNVs per patient (Min, 25%, 75%, Max)
CRLMM/VanillaIce	45,757	7,958	37,799	30 (4, 21, 42, 438)

PennCNV	50,908	16,132	34,776	39 (13, 31, 48, 96)

Concordance of detected CNV regions by PennCNV and CRLMM/VanillaIce was also evaluated. Again, we limited the comparison to the 1,266 subjects that had CNV calls using both software packages and passed the CNV-specific quality control metrics that were provided by PennCNV and the signal-to-noise metric suggested by CRLMM. Table 4 provides the percent of concordant loci per sample; a locus is defined to be concordant if both PennCNV and CRLMM/VanillaIce identified a deletion/duplication that contained the corresponding locus. Concordance was defined with respect to regions identified as duplicated or deleted and not with respect to the actual copy number state. Across all loci contained within regions that were identified to be duplicated, the median concordance between PennCNV and CRLMM/VanillaIce was 47.9%. Similarly, across all loci contained within regions that were identified to be deleted, the median concordance between PennCNV and CRLMM/VanillaIce was 51.5%. The fact that CRLMM/VanillaIce identifies segments that consist of a single locus and PennCNV requires at least three loci does not affect these results to any large extent since only 0.7% of the segments detected by CRLMM/VanillaIce consist of fewer than three loci. Although the agreement seems to be poor (i.e., 50:50), it agrees with a previous publication comparing software packages that were designed for the 100k Affymetrix SNP array [2]. Baross and colleagues [2] reported that 63% of duplications and 37% of deletions were detected by two or more software packages. Interestingly, PennCNV detects almost all of the regions that CRLMM/VanillaIce does as well as additional regions of copy number gain and loss (Table 4).

**Table 4:** Concordance of detected CNV segments using PennCNV and CRLMM/VanillaIce for 1,266 subjects that passed PennCNV and CRLMM QC metrics

	Quantile				
	**0% (Min)**	**25%**	**50% (Median)**	**75%**	**100% (Max)**

Duplications					

Concordance	0.0	26.3	47.9	68.2	100.0

PennCNV only	0.0	24.7	44.9	67.9	100.0

CRLMM/VanillaIce only	0.0	0.3	2.1	7.1	100.0

					

Deletions					

Concordance	0.4	37.5	51.5	65.5	95.8

PennCNV only	0.0	23.0	37.5	53.6	94.0

CRLMM/VanillaIce only	0.0	2.3	5.0	11.8	96.7

**Figure 5:**
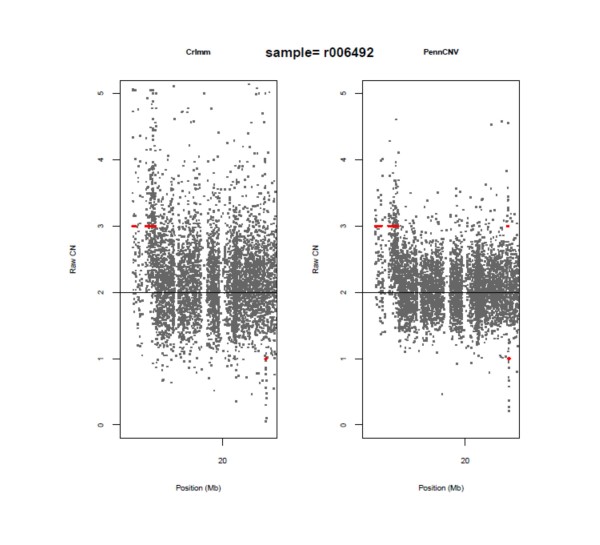
**A 8 Mb region on chromosome 12 displaying CNVs identified by CRLMM/VanillaIce and PennCNV for subject r006492**. Dots denote the locus-level copy number data and the vertical red lines denote regions of identified CNVs.

**Figure 6:**
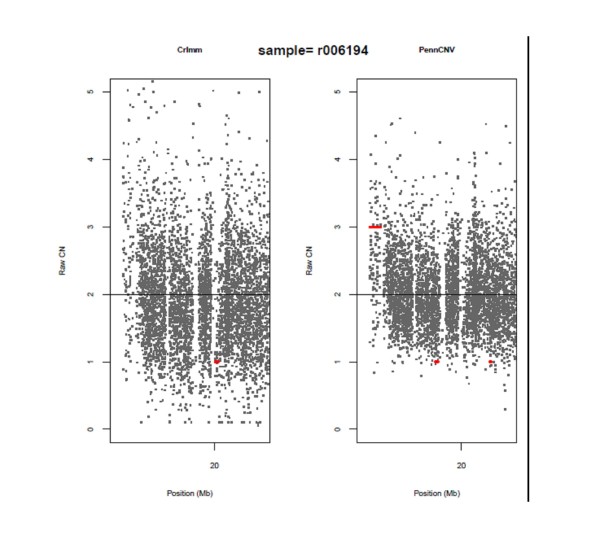
**The same 8 Mb region on chromosome 12 displayed in Figure 5, but for subject r006194**. Dots denote the locus-level copy number data and the vertical red lines denote regions of identified CNVs.

The discordance between PennCNV and CRLMM/VanillaIce can be largely attributed to the variability of the locus-level copy number data obtained from PennCNV and CRLMM. Figure 5 displays three regions on chromosome 22 that are concordant between PennCNV and CRLMM/VanillaIce; the locus-level data are denoted by black dots and the identified segments are denoted by red horizontal lines. Although the locus-level copy number data are clearly more variable for CRLMM in comparison to PennCNV, the software identified three common regions: two regions of amplification and one deleted region. Additionally, PennCNV detected a small region of amplification at ~22.6 Mb that CRLMM/VanillaIce did not likely due to the variability associated with the CRLMM locus-level copy number data. As another example, Figure 6 displays the same region on chromosome 22 as Figure 5, but for a different subject. Again, PennCNV identified a region of amplification that appears to be valid from visual inspection whereas CRLMM/VanillaIce was unable to identify the region above the noise in the data. There are two deleted regions detected by PennCNV and one deleted region detected by CRLMM/VanillaIce that are discordant, but from visual inspection it is difficult to determine if these regions are real or in fact false positives.

### False-Positive Rate

**Figure 7:**
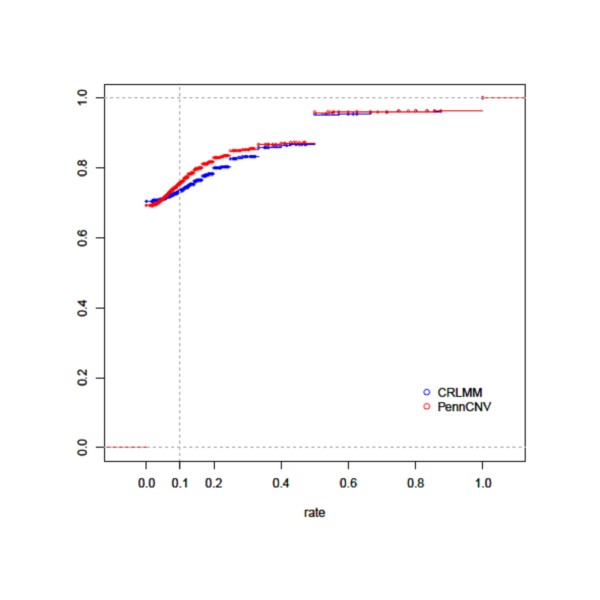
**Cumulative density of the rate of heterozygous SNPS as obtained from PennCNV and CRLMM/VanillaIce**.

**Figure 8:**
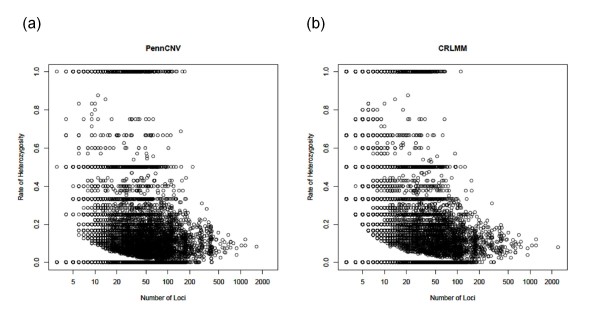
**Relationship between segment size and the rate of heterozygous SNPS**. for segments obtained from (a) PennCNV and (b) CRLMM/VanillaIce.

Ultimately, it is important to know what proportion of the detected segments are false positives for both PennCNV and CRLMM. To do so, we took the approach described by Baross et al. [2] and assumed that deletions should not contain heterozygous genotype calls (see Methods). Thus, we evaluated the 37,799 deletions detected by CRLMM and 34,776 deletions detected by PennCNV and compared the estimated false-positive rate amongst the two software. Baross and colleagues [2] defined a deletion as a false positive if the rate of heterozygous SNPs was more than 10% of the total SNP count. Figure 7 displays the cumulative density of the rate of heterozygous SNPs across all autosomes for PennCNV and CRLMM; both hemizygous and homozygous deletions were included. Using a 10% threshold, as suggested by Baross and colleagues [2], 26% of the 37,799 deletions detected by CRLMM are probable false positive regions whereas approximately 24% of the 34,776 deletions detected by PennCNV are probable false positive regions. This implies that the extra regions detected by PennCNV are not likely to be false positives. Additional File 1 provides the heterozygous rate for each of the 22 autosomes individually and shows that the false-positive rate differs across the autosomes. Figure 8 displays the relationship between the size of each detected deleted segment (number of loci) and the estimated rate of heterozygous SNPs. To note, deleted segments that have 100% heterozygous rate include both hemizygous and homozygous deletions.

## Conclusions

Our objective was to compare commonly-used freely-available software algorithms for analyzing CNV data obtained from the Affymetrix 6.0 SNP array platform. Specifically, we compared Affymetrix Power Tools (APT), Aroma.Affymetrix, PennCNV and CRLMM. Of the four software packages that we compared APT performed the best with respect to bias; that is, APT on average had median locus-level copy numbers closest to a value of two. In fact, the tight bounds associated with the bias for APT suggests that there could be an algorithmic assumption made by the software; however, we could not identify any such discussion in their documentation. Although APT had the smallest bias, PennCNV and Aroma.Affymetrix had the smallest variability associated with the median locus-level copy number. Thus, if one is interested in performing statistical tests on the locus-level copy number data, our empirical results suggest that PennCNV and Aroma.Affymetrix are the optimal software packages of those evaluated herein.

Batch effects are an artefact of processing samples in multiple laboratories, by multiple technicians, using reagents from multiple batches, or other sample processing steps that are not constant across samples and affects individual probes differently [1]. Methodologies to remove probe-specific batch effects are an area of active research. Here, we have shown empirically that the extent of probe-specific batch effects post analytical processing is dependent on the software used. Furthermore, even though CRLMM has a default option to remove batch effects, probe-specific batch effects were present in the post-processed locus-level copy number data. Additionally, probe-specific batch effects were more evident in the post-processed CRLMM and PennCNV locus-level copy number data in comparison to the Aroma.Affymetrix and APT post-processed data. This empirical observation was surprising and thus warrants additional evaluation.

It is interesting - and maybe surprising - that so many deleted segments were estimated to be false positives, especially since both PennCNV and VanillaIce utilize genotype and B-allele frequency information in their HMM algorithms. Even though our estimated false-positive rate appears high, our results agree with the rates shown by Baross et al. [2]. Undoubtedly, the gold standard would be to have known-spike in data in which to compare software and likewise determine the true false-positive rate or to validate each candidate CNV region using an independent technology. Unfortunately, validation is not feasible for the thousands of candidate CNVs identified in the GENOA data.

Of the software evaluated, only PennCNV and CRLMM (through the use of VanillaIce, which was developed by the same authors) provide locus-level copy number data as well as a segmentation routine and thus allow the analyst to complete all data processing steps without having to reformat the data for use in another software package. PennCNV provides CNV-specific quality-control metrics to aid in identifying potentially poor CNV samples, whereas CRLMM or VanillaIce does not. That is, CRLMM suggests using a signal-to-noise measure to identify samples that have poor quality for genotype calling but do not have additional CNV-specific quality-control measures. We observed that PennCNV detects almost all of the regions that CRLMM/VanillaIce does as well as additional regions of copy number gain and loss. Although some of these additional regions may be false positives, the estimated false-positive error rate associated with deletions was similar for PennCNV and CRLMM/VanillaIce and thus the additional regions are likely not all false positives.

As discussed by Lai et al. [15], it is very difficult to compare complicated algorithms as each algorithm has its own set of parameters that must be optically tuned. Therefore, it is possible that the results discussed herein would change if the parameters associated with the software were fine tuned to fit the data more precisely. Unfortunately, there is little-to-no guidance provided by most software for evaluating and choosing optimal parameter settings. This was particularly true for the HMM algorithms provided by PennCNV and VanillaIce. Thus, we evaluated each algorithm using the default parameters. Although not optimal, this is what many analysts - even experienced analysts - will ultimately do until developers of software provide adequate documentation and guidance for evaluating and choosing parameters for complicated algorithms. Until such guidance exists, we recommend trying multiple algorithms, evaluating concordance/discordance as we have done here and subsequently consider the union of regions for downstream association tests. Others have suggested a similar approach [2,3] assuming that algorithms have different strengths and thus taking the union of all the copy number aberrations should increase sensitivity.

## Methods

### Affymetrix 6.0 SNP array: GENOA Data

The samples included 1,418 of the non-Hispanic white adults enrolled in the Genetic Epidemiology Network of Arteriopathy (GENOA) study of the Family Blood Pressure Program (FBPP), a study designed to identify germline genetic determinants of hypertension in multiple ethnic groups. These samples were genotyped using the Affymetrix SNP Array 6.0 and all had contrast QC values greater than 0.4.

### PennCNV

Copy number analysis was performed using PennCNV (Version 2010May01) software using the default parameters [4]. Locus-level copy number for sample *i *and probe *j *is calculated as Log_2 _R_ij _= log_2 _(R_observed_/R_expected_), where R_*observed *_is the summation of the intensity for the A and B allele at probe *j *for sample *i *and R_expected _for probe *j *is computed from linear interpolation of canonical genotype clusters [4]. The  transformation was applied for plotting purposes and for calculating bias and variance. Affymetrix Power Tools (APT) was used to obtain genotype calls; the genotype calls are required for PennCNV copy number estimation. Regions of copy number aberrations were identified using the hidden Markov model (HMM) algorithm provided within PennCNV. The PennCNV-Affy Protocol (http://www.openbioinformatics.org/penncnv/penncnv_tutorial_affy_gw6.html) was followed to obtain the Log_2_R and B-allele frequency (BAF) values. PennCNV provides CNV-specific quality-control metrics for which to identify potentially poor CNV samples. Subjects were excluded if they had more than 100 CNV intervals detected, B-allele drift > 0.0125, wave factor > 0.05, or Log_2_R standard deviation > 0.40. The wave factor represents the overall waviness or variation of signal intensity and the B-allele drift is the fraction of "abnormal" markers that do not cluster in the usual positions (0, 0.5, 1); this number is the median of all chromosomes. In some cases when a portion of the array has genotyping failure (for example, a corner of the array is dried), most other markers will look normal but some markers will appear very random; the B-allele drift is useful for detecting these situations (personal correspondence with PennCNV). Additional File 2 provides the code used to run PennCNV on the GENOA data.

### Aroma.Affymetrix

Copy number analysis was performed using Aroma.Affymetrix (Version 1.3.0) software using the default parameters (http://www.aroma-project.org) [6]. Locus-level copy number for sample *i *and probe *j *is calculated as , where  is the normalized total copy number and  is a reference signal at probe *j *typically representing the mean diploid signal. The  transformation was applied for plotting purposes and for calculating bias and variance. Additional File 2 provides the code used to run Aroma.Affymetrix on the GENOA data.

### Affymetrix Power Tools (APT)

Copy number analysis was performed using Affymetrix Power Tools (Version 1.12.0) software using the default parameters [7]. Locus-level copy number for sample *i *and probe *j *is calculated as , where  is the normalized total copy number and  is a reference signal at probe *j *typically representing the mean diploid signal. The  transformation was applied for plotting purposes and for calculating bias and variance. Additional File 2 provides the code used to run APT on the GENOA data.

### CRLMM

Copy number analysis was performed using CRLMM (Version 1.4.3) software using the default parameters [1]. CRLMM estimates absolute copy number using a linear model that can be summarized as I = O + NS + S, where I denotes the observed intensity, O denotes optical background, NS denotes nonspecific hybridization and S denotes the change in the average intensity at a given locus per each integer increase in the allelic copy number. Absolute copy number is then estimated as ; please see [1] for specifics. Batch was defined according to the 96-well plate the sample was processed on. Regions of copy number aberrations were identified using the HMM algorithm provided by VanillaIce (Version 1.6.0) [12]. Although CRLMM does not itself perform segmentation, the copy number data produced by CRLMM can be directly imported into VanillaIce since they were developed by the same authors. Subjects were excluded if they had a signal-to-noise measure larger than 0.40. Additional File 2 provides the code used to run CRLMM and VanillaIce on the GENOA data.

### Statistical Methods

The median locus-level copy number was computed across all loci and chromosomes for each sample. Because these are germline data, we expect a median copy number of two representing no change. Bias and variance were evaluated on the locus-level data across all chromosomes. Bias was calculated as the median raw copy number across all probes minus two. To evaluate variability, the median absolute deviation (MAD) and derivative log ratio spread (DLRS) was computed across all loci for each sample and chromosome.

To evaluate the agreement across the four software packages Bland-Altman plots were utilized [11]. A Bland-Altman plot is a plot of the difference between two measurements (X - Y) against the average of the two measurements (X + Y)/2. In comparison to a simple correlation plot of X versus Y, a Bland-Altman plot provides a better visualization of the magnitude of disagreement (error and bias) and better highlights outliers and trends in the disagreement. If the differences in locus-level copy number between two software packages are not related to the magnitude of either copy number measurement, then it is expected that the data will be randomly scattered around the zero horizontal reference line.

Concordance of detected CNV regions by PennCNV and CRLMM/VanillaIce was evaluated on a locus-level basis, separately for deletions and duplications. Let *i *denote subject, *j *denote locus and *k *denote software (here *k *= 1, 2). We calculated the locus-specific concordance as(1)

Where *y*_*ijk *_is an indicator that locus *j *was included in a region identified as a deletion/duplication by software *k *for subject *i*. Thus, *X*_*ij *_is equal to 0 if locus *j *was not in a region identified by either software as a deletion/duplication, 1 if locus *j *was identified by only one of the software as a deletion/duplication and 2 if locus *j *was identified by both software packages as a deletion/duplication. To evaluate subject-specific concordance across PennCNV and CRLMM/VanillaIce, we calculated(2)

where  is an indicator function for loci that were detected using both software algorithms and similarly, is an indicator function for loci that were detected by at least one of the software algorithms. Here, we are evaluating concordance of two different algorithms on the exact same sample and thus we calculated concordance on a locus basis instead of on a segment basis. Even though we calculated concordance on locus-level data, we obtained similar levels of concordance that others have reported with respect to segmented-level data [2].

## Authors' contributions

JEEP and EJA designed and coordinated the study, helped to perform the statistical analyses and drafted the manuscript. SM and EJA performed the statistical analyses. MdA participated in the design of the study, helped to draft the manuscript, and obtained funding. SLRK participated in the data collection and obtained funding. All authors read and approved the manuscript.

## Supplementary Material

Additional file 1 **Cumulative density of the rate of heterozygous SNPS for each of the autosomes individually as obtained from PennCNV and CRLMM/VanillaIce**. Click here for file

Additional file 2 **Software settings for Aroma.Affymetrix, Affymetrix Power Tools (APT), PennCNV and CRLMM/VanillaIce**. Click here for file
