# Supplementary material for: Software comparison for evaluating genomic copy number variation for Affymetrix 6.0 SNP array platform
Source: BMC Bioinformatics. 2011 May 31;12:220. doi: 10.1186/1471-2105-12-220 (PMC3146450; doi:10.1186/1471-2105-12-220)
Supplement: Additional file 1 — Cumulative density of the rate of heterozygous SNPS for each of the autosomes individually as obtained from PennCNV and CRLMM/VanillaIce. [file 1471-2105-12-220-S1.PDF]

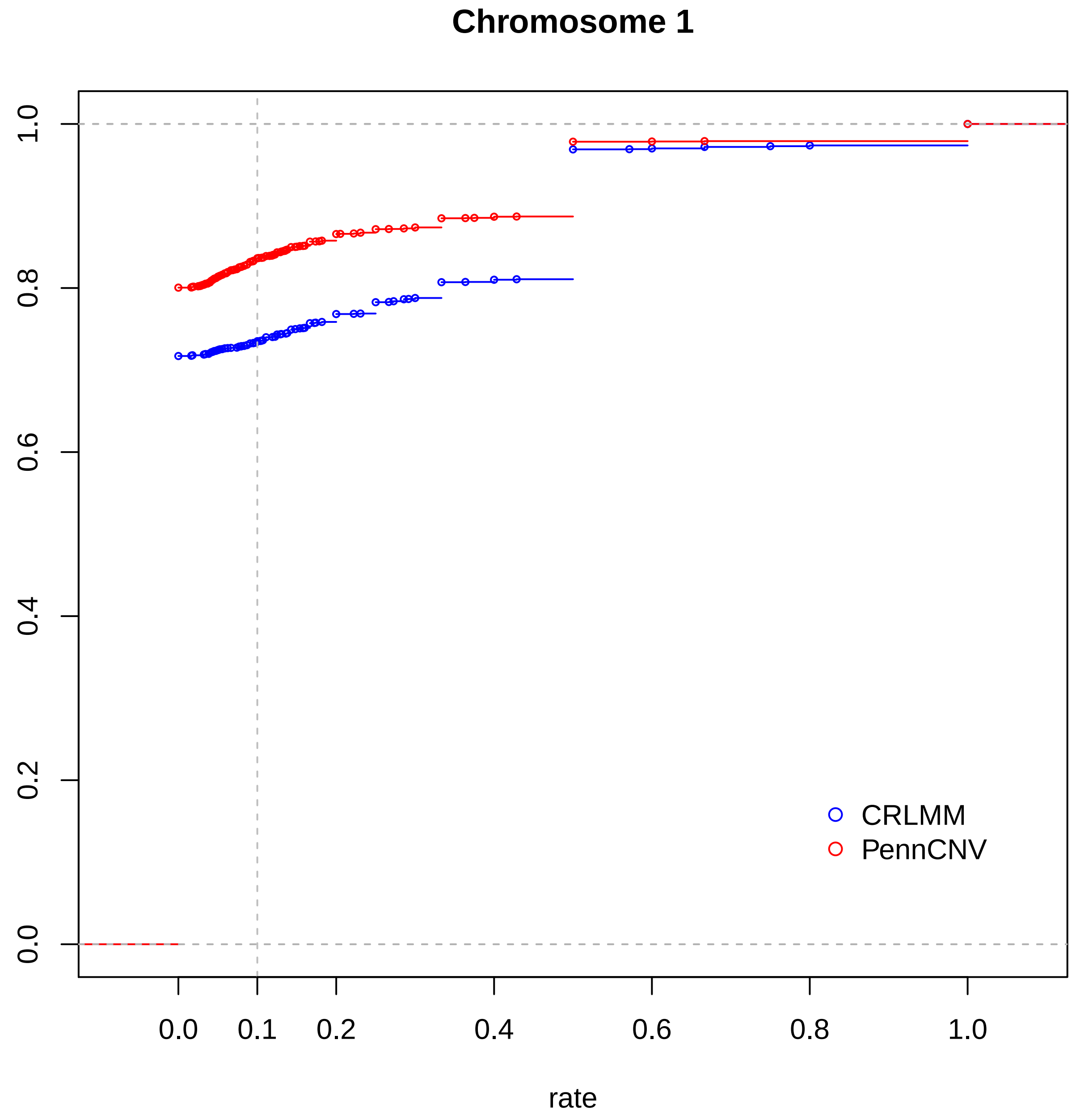
Chromosome 1
1.0
0.8
0.6
0.4
0.2
0.0
0.0
0.1
0.2
0.4
0.6
0.8
1.0
rate
CRLMM
PennCNV

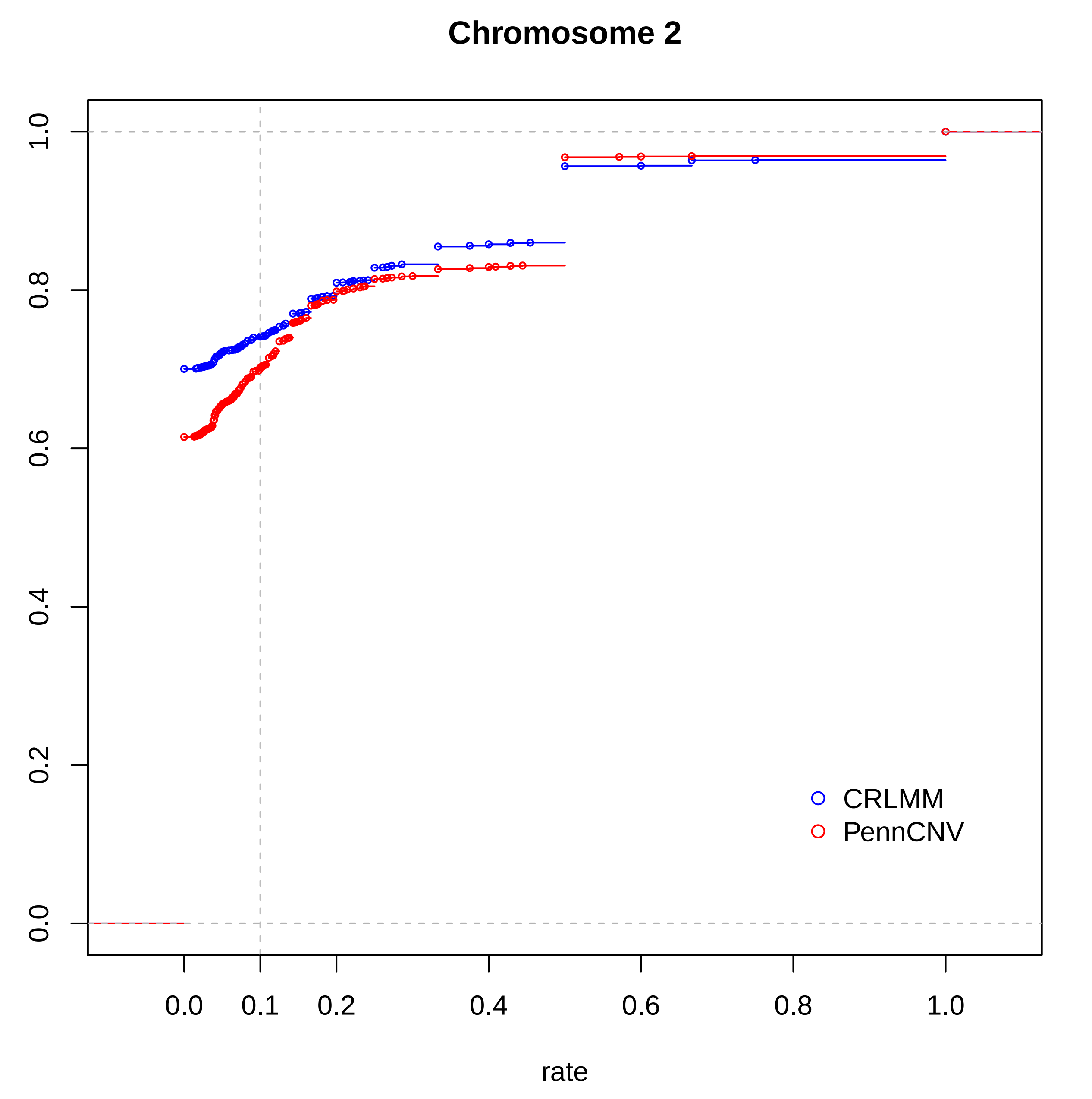

Chromosome 2
0.0
0.2
0.4
0.6
0.8
1.0
0.0
0.1
0.2
0.4
0.6
0.8
1.0
rate
CRLMM
PennCNV

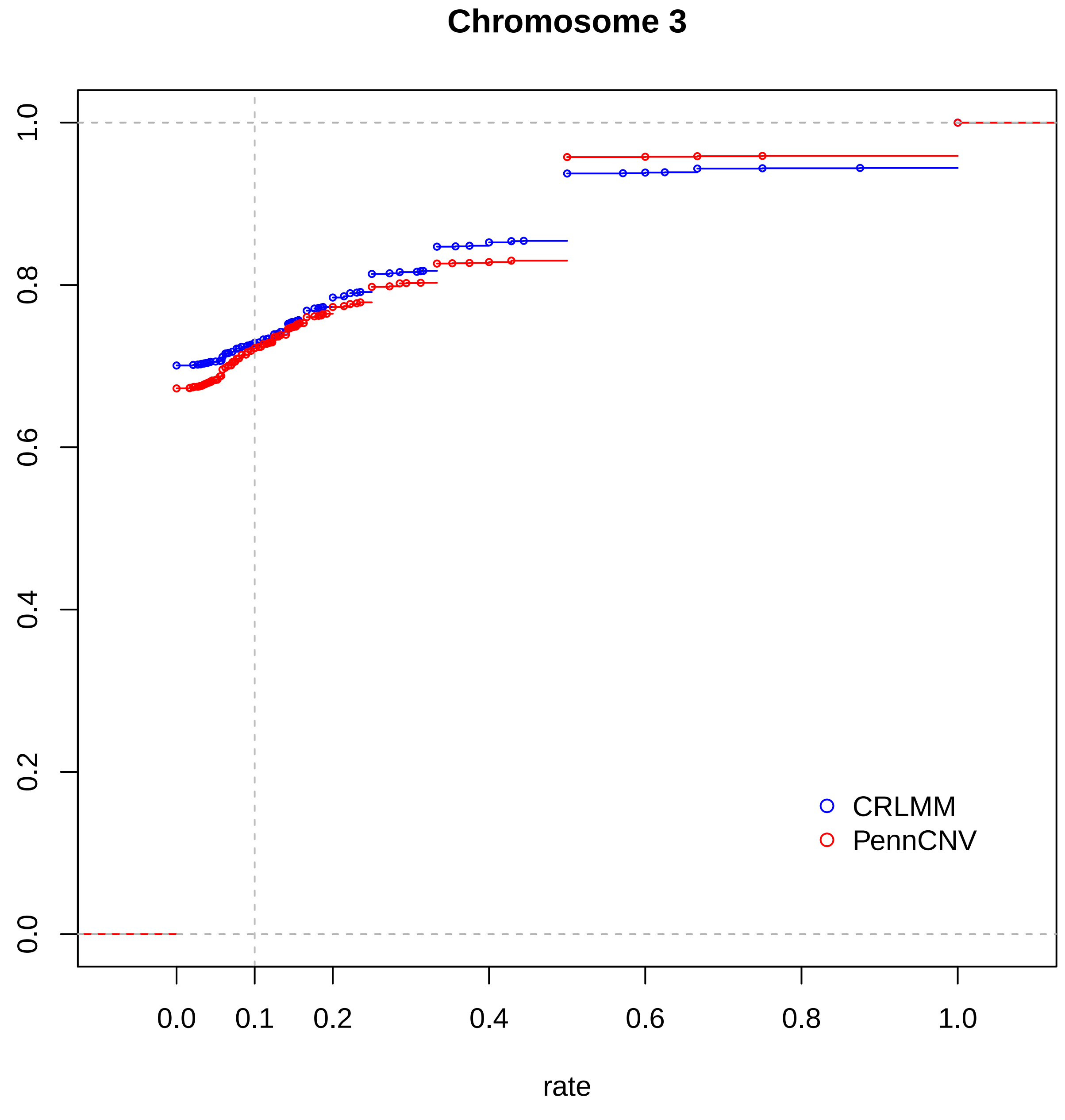

Chromosome 3
1.0
0.8
0.6
0.4
0.2
0.0
0.0
0.1
0.2
0.4
0.6
0.8
1.0
rate
CRLMM
PennCNV

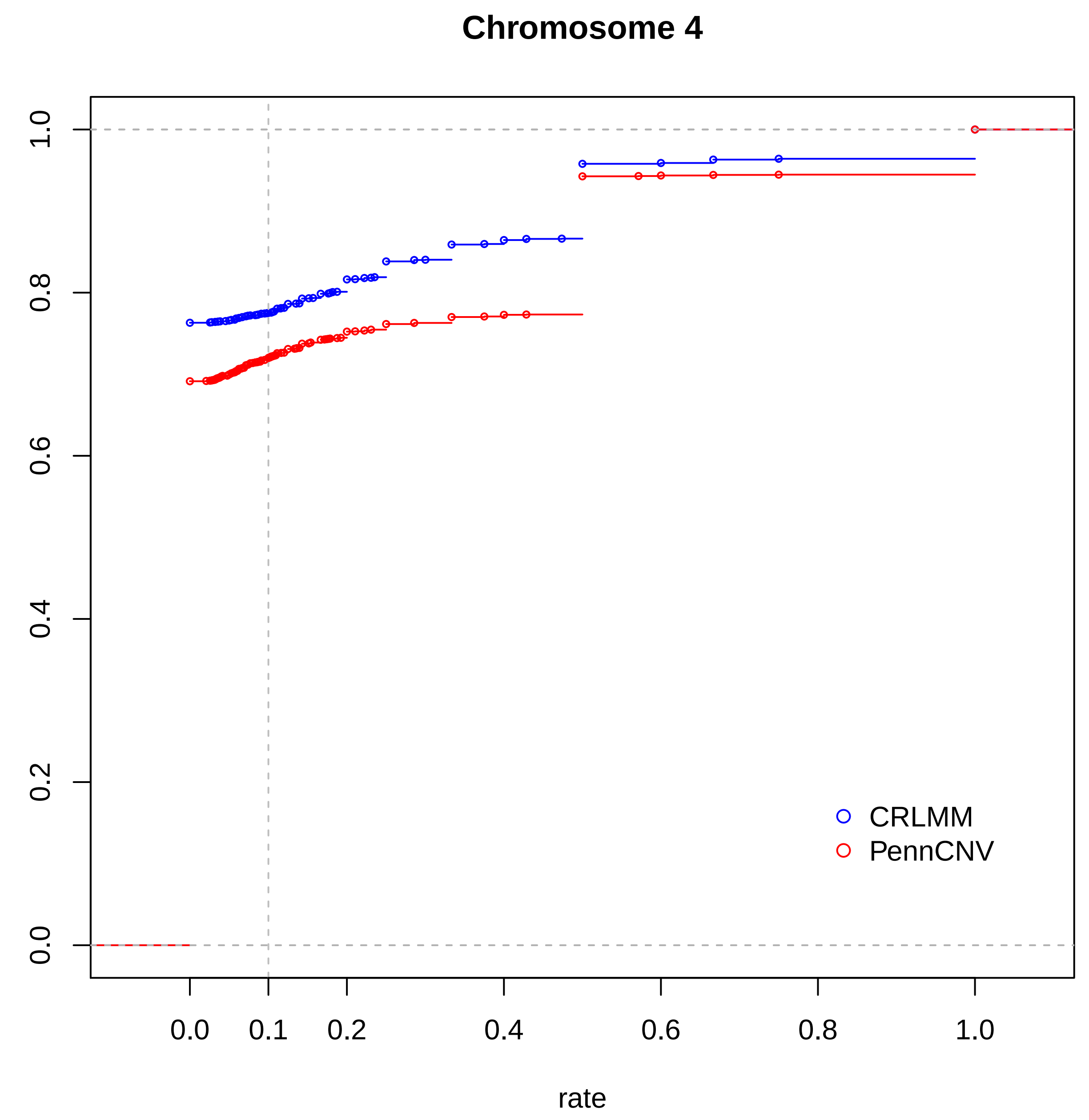

Chromosome 4
1.0
0.8
0.6
0.4
0.2
0.0
0.0
0.1
0.2
0.4
0.6
0.8
1.0
rate
CRLMM
PennCNV

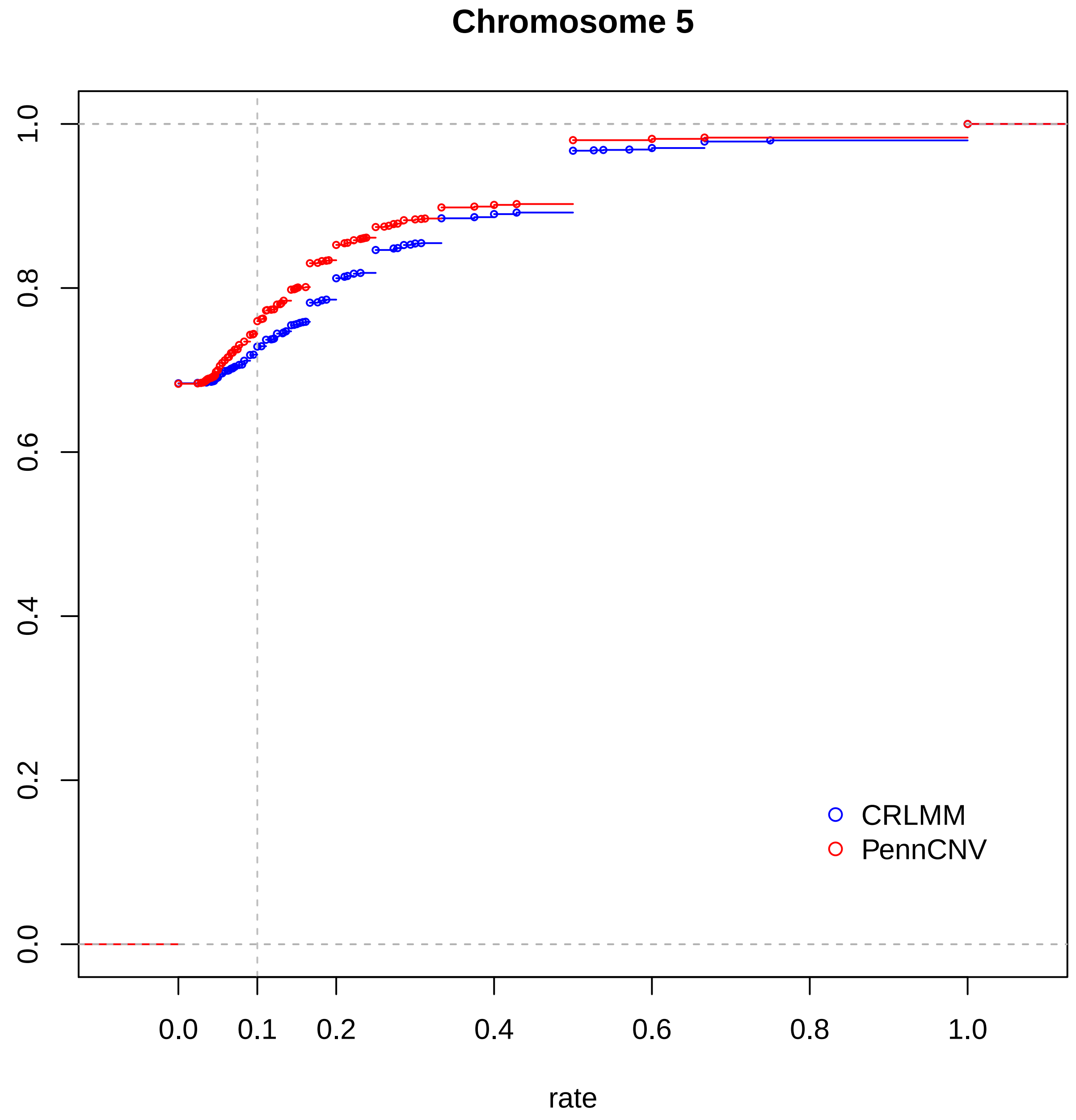
Chromosome 5
1.0
0.8
0.6
0.4
0.2
0.0
0.0
0.1
0.2
0.4
0.6
0.8
1.0
rate
CRLMM
PennCNV

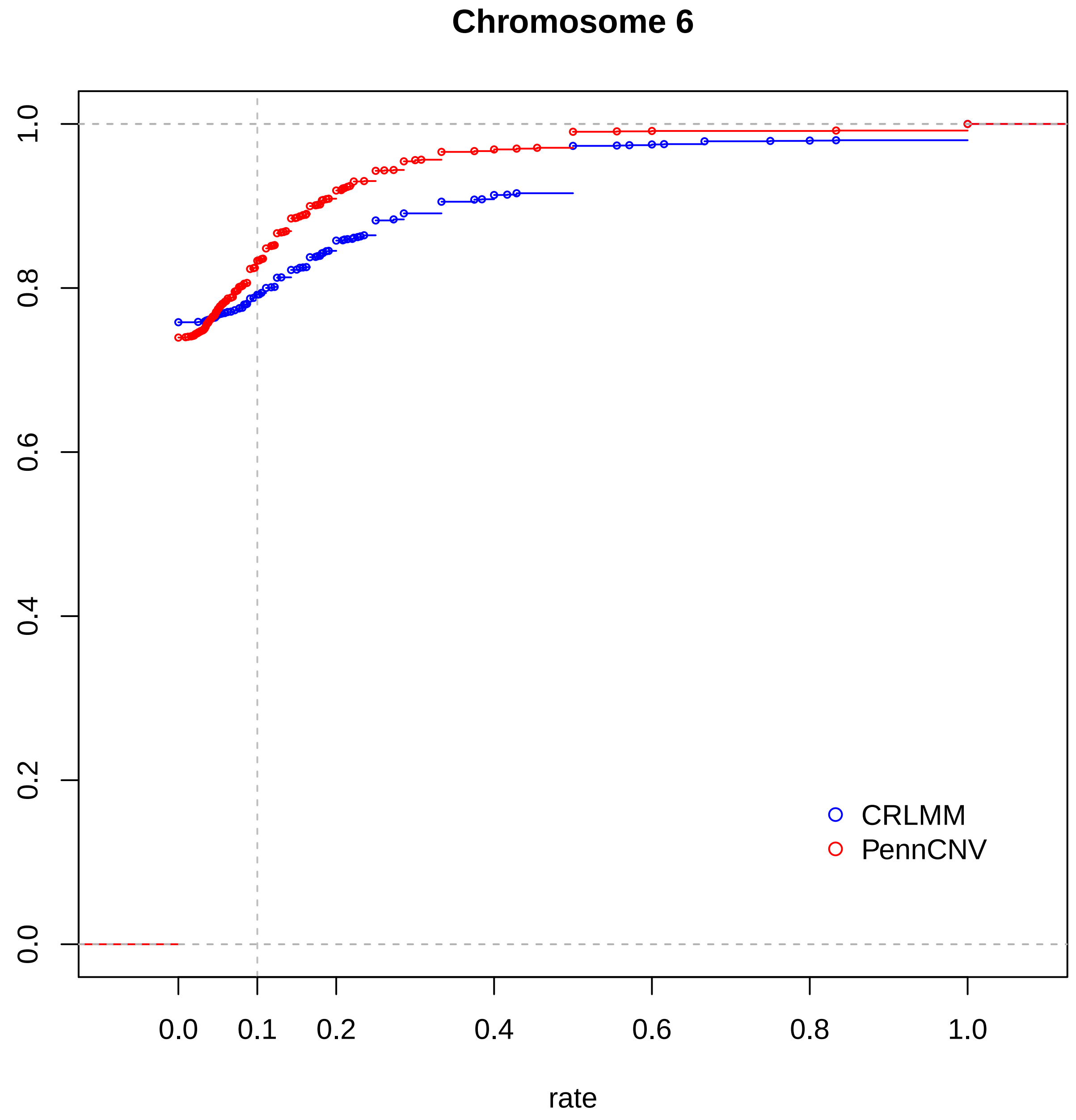

Chromosome 6
1.0
0.8
0.6
0.4
0.2
0.0
0.0
0.1
0.2
0.4
0.6
0.8
1.0
rate
CRLMM
PennCNV

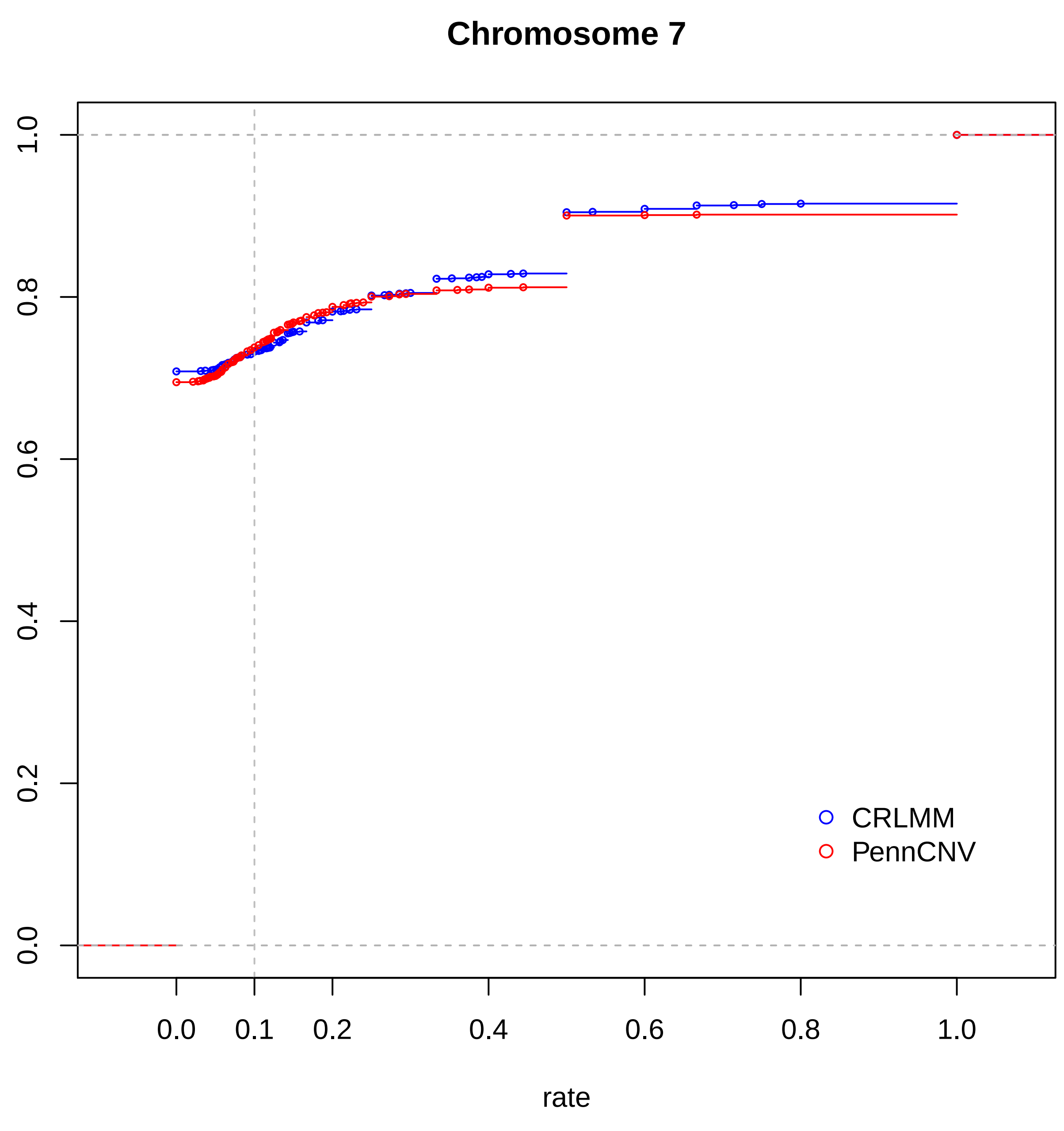
Chromosome 7
0.0
0.2
0.4
0.6
0.8
1.0
0.0
0.1
0.2
0.4
0.6
0.8
1.0
rate
CRLMM
PennCNV

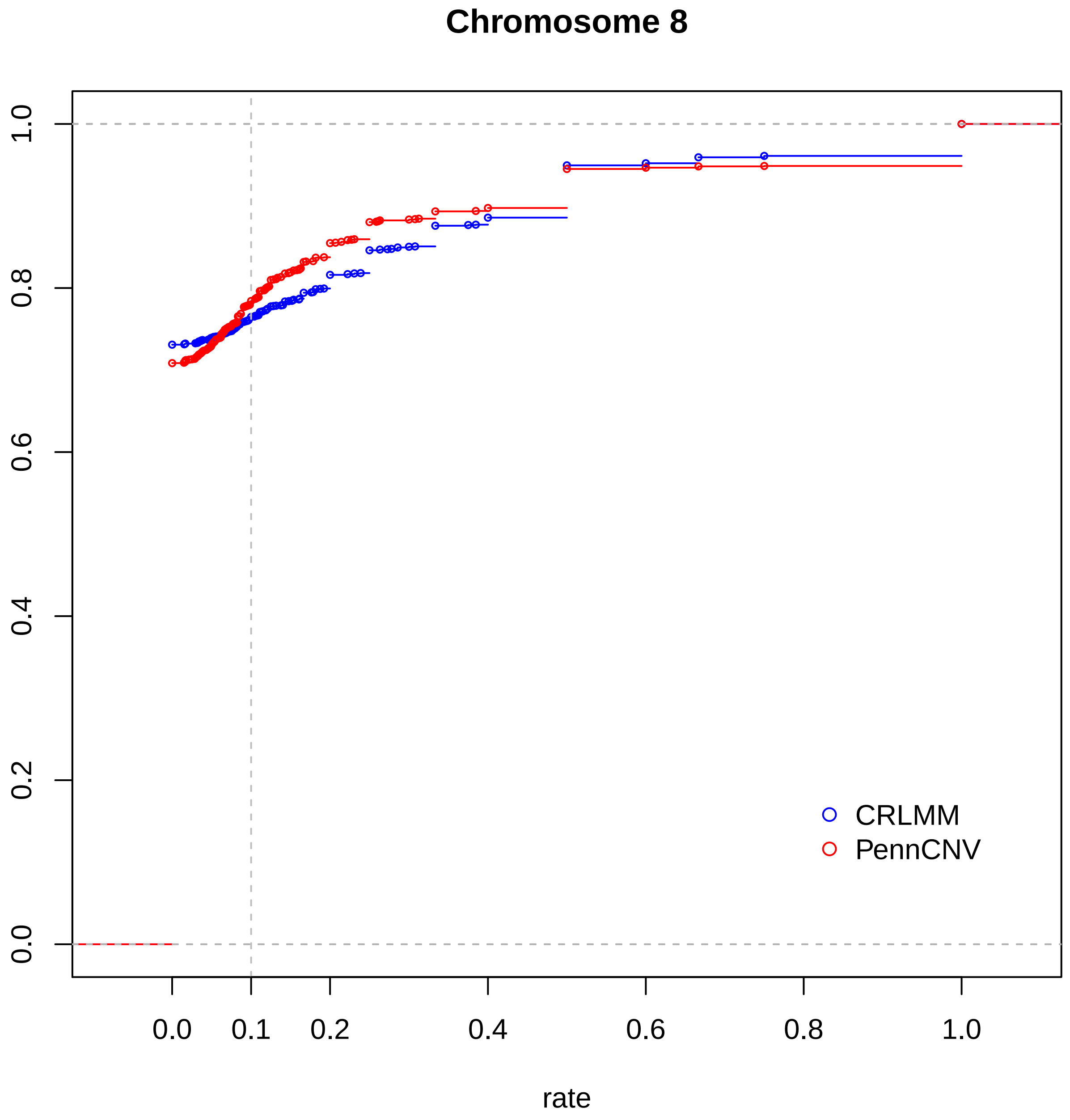

Chromosome 8
1.0
0.8
0.6
0.4
0.2
0.0
0.0
0.1
0.2
0.4
0.6
0.8
1.0
rate
CRLMM
PennCNV

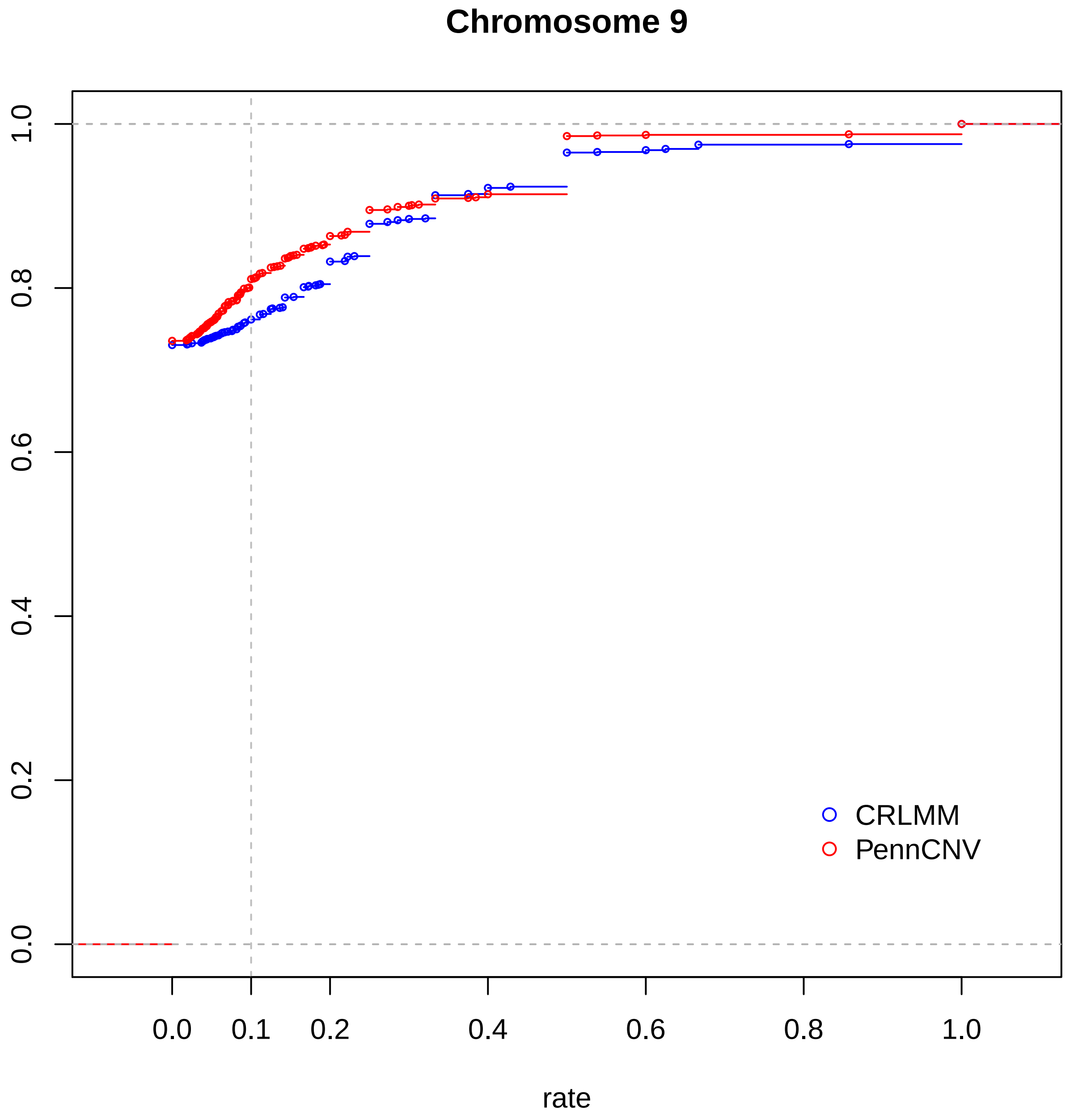
Chromosome 9
0.0
0.2
0.4
0.6
0.8
1.0
0.0
0.1
0.2
0.4
0.6
0.8
1.0
rate
CRLMM
PennCNV

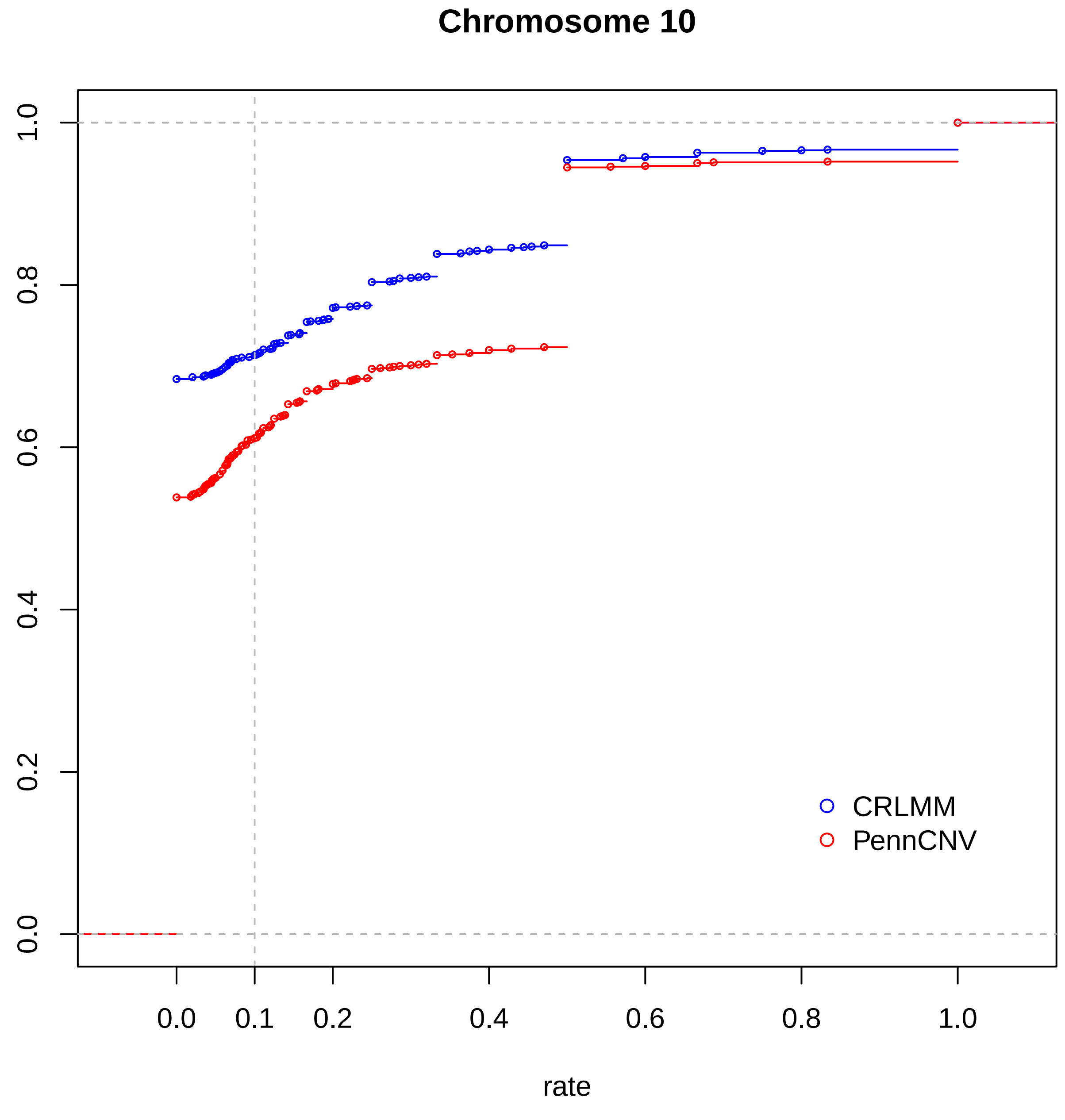

Chromosome 10
1.0
0.8
0.6
0.4
0.2
0.0
0.0
0.1
0.2
0.4
0.6
0.8
1.0
rate
CRLMM
PennCNV

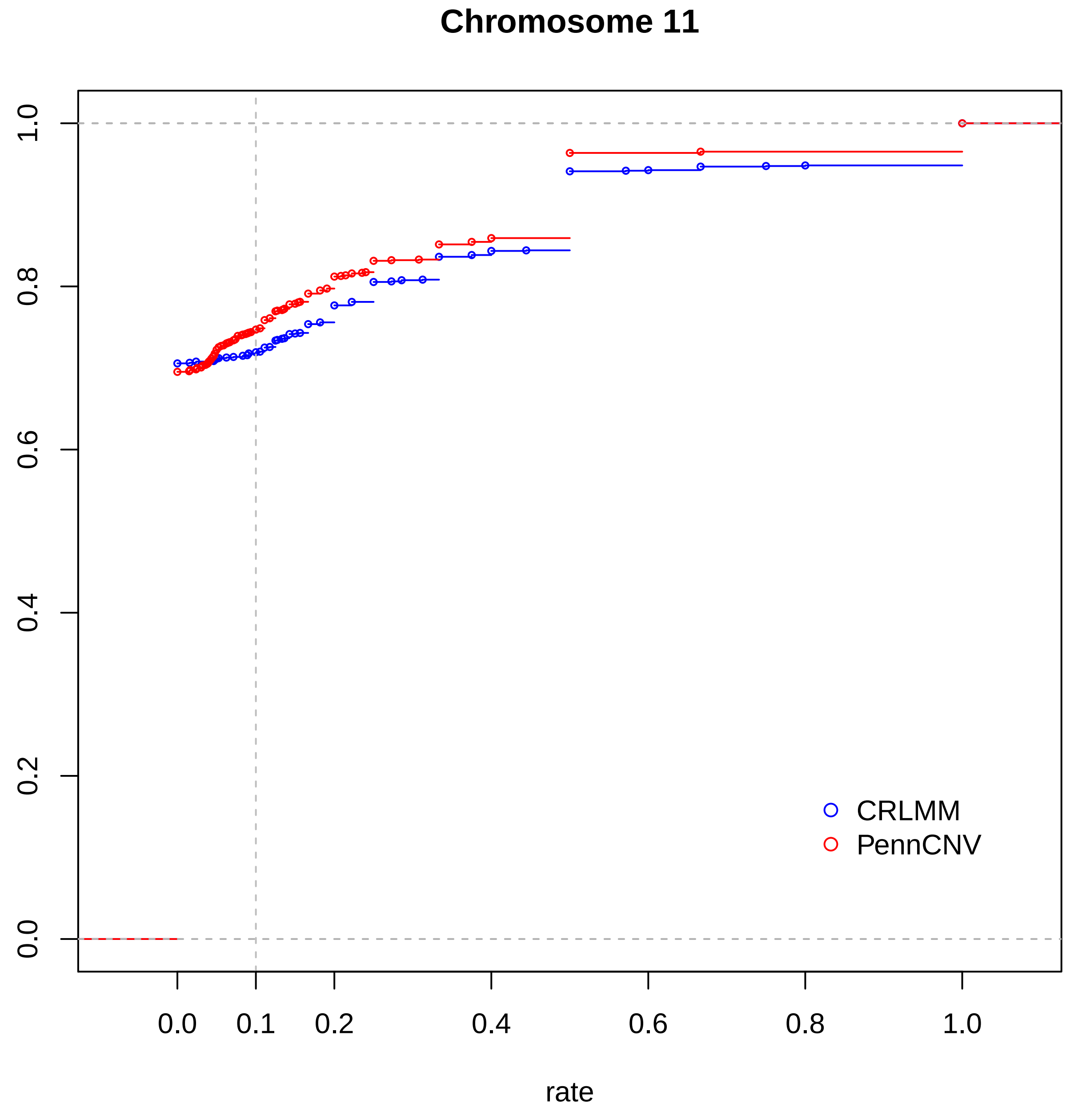
Chromosome 11
1.0
0.8
0.6
0.4
0.2
0.0
0.0
0.1
0.2
0.4
0.6
0.8
1.0
rate
CRLMM
PennCNV

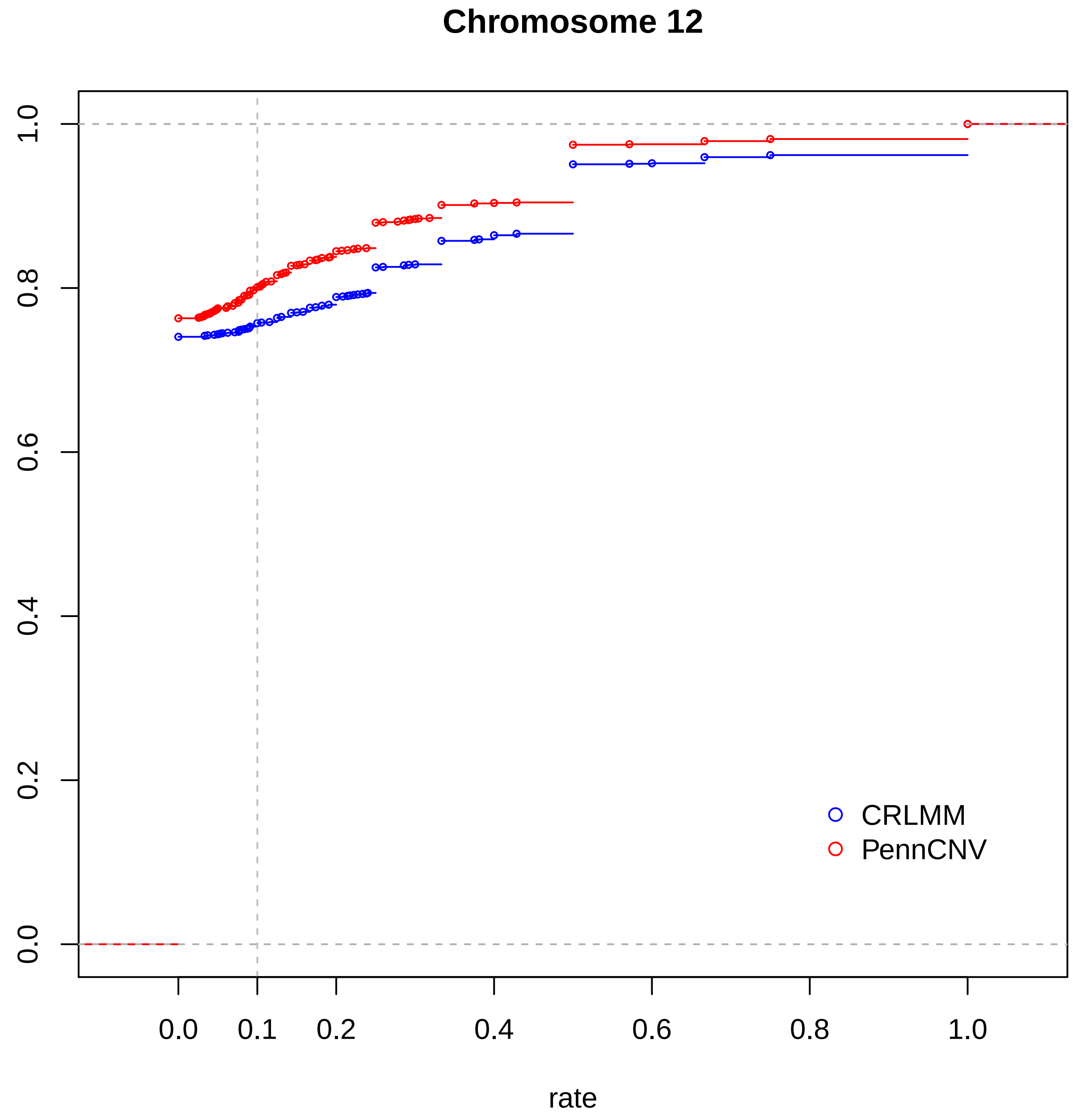
Chromosome 12
1.0
0.8
0.6
0.4
0.2
0.0
0.0
0.1
0.2
0.4
0.6
0.8
1.0
rate
CRLMM
PennCNV

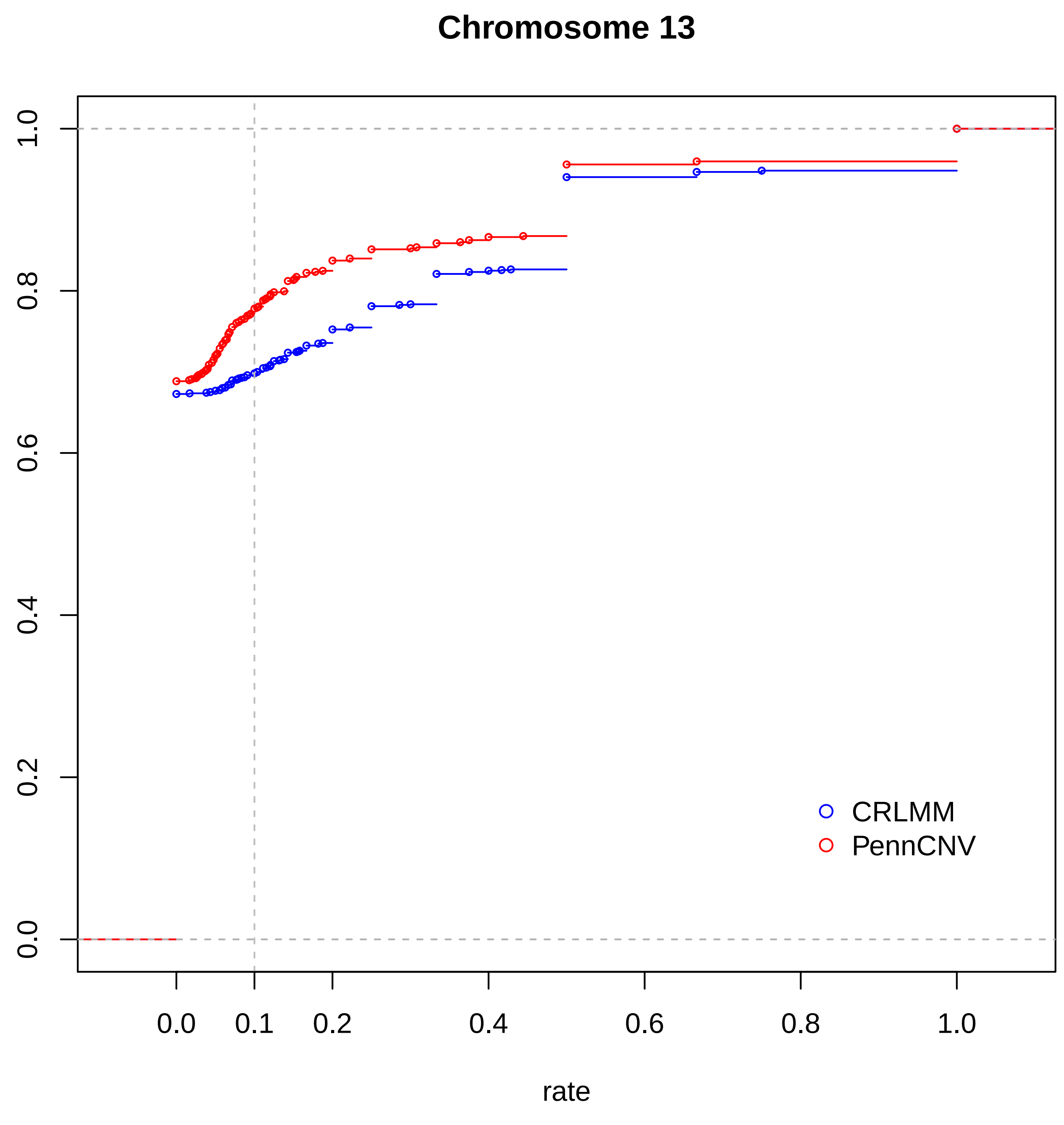

Chromosome 13
1.0
0.8
0.6
0.4
0.2
0.0
0.0
0.1
0.2
0.4
0.6
0.8
1.0
rate
CRLMM
PennCNV

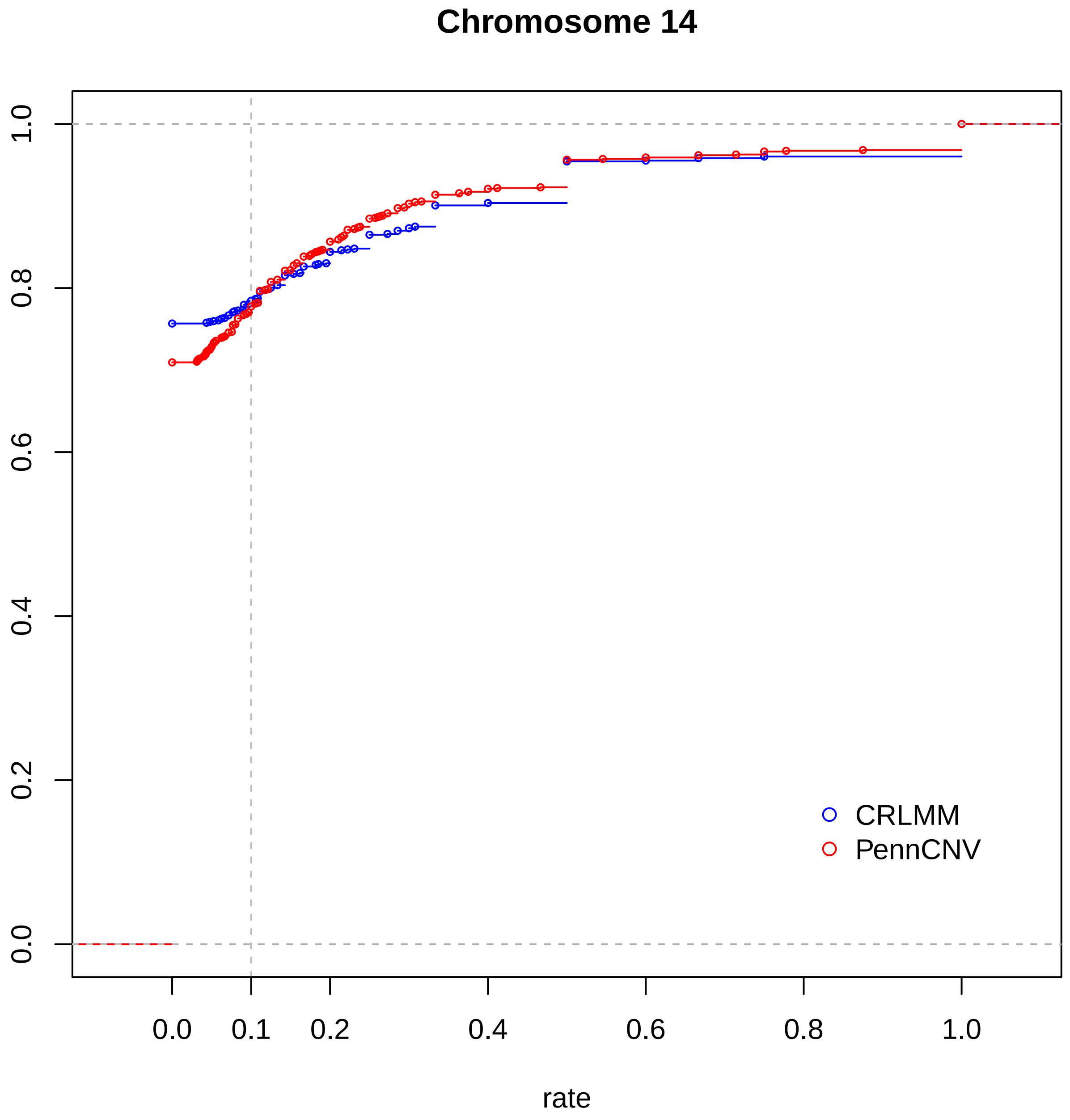
Chromosome 14
1.0
0.8
0.6
0.4
0.2
0.0
0.0
0.1
0.2
0.4
0.6
0.8
1.0
rate
CRLMM
PennCNV

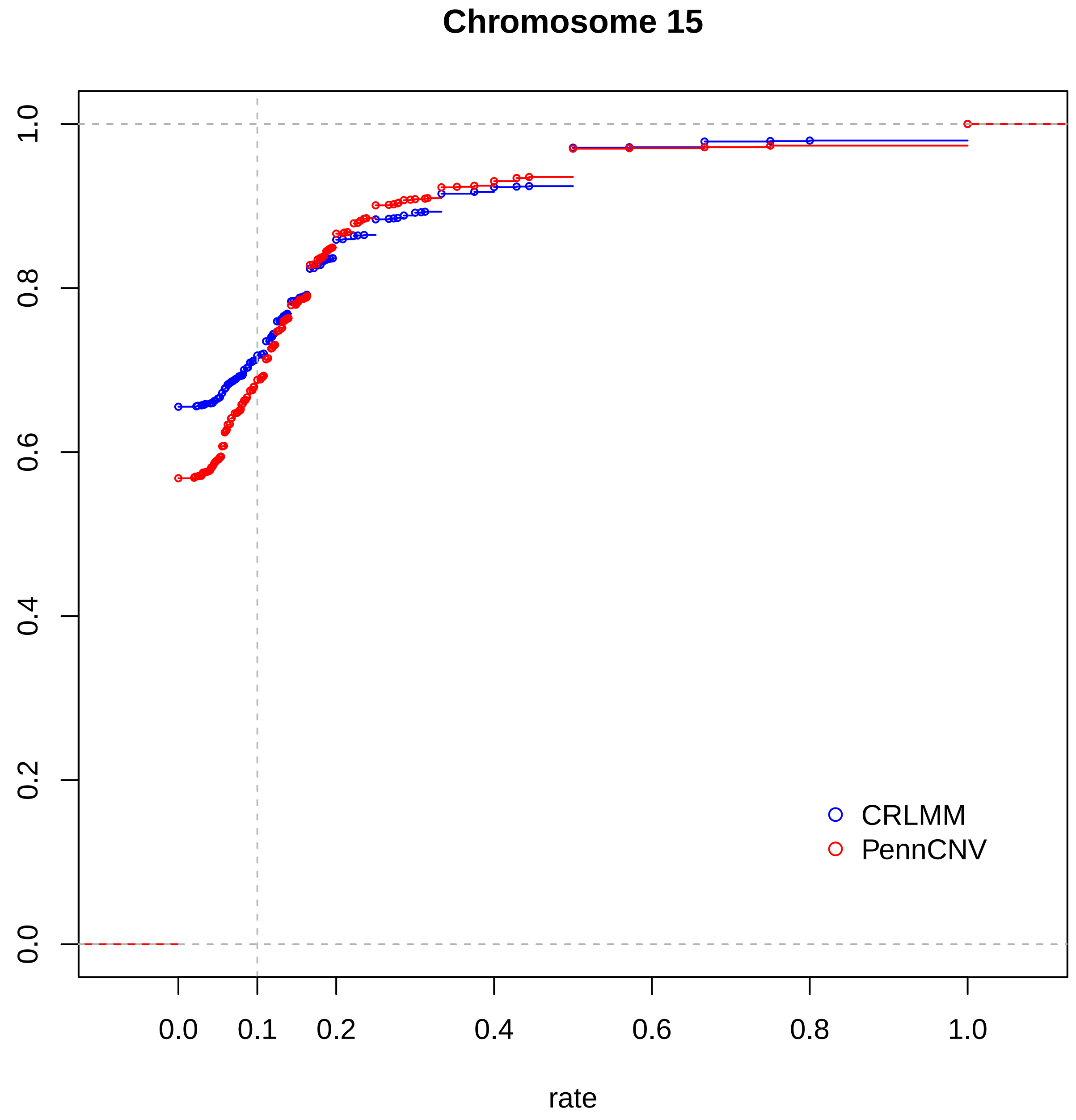

Chromosome 15
1.0
0.8
0.6
0.4
0.2
0.0
0.0
0.1
0.2
0.4
0.6
0.8
1.0
rate
CRLMM
PennCNV

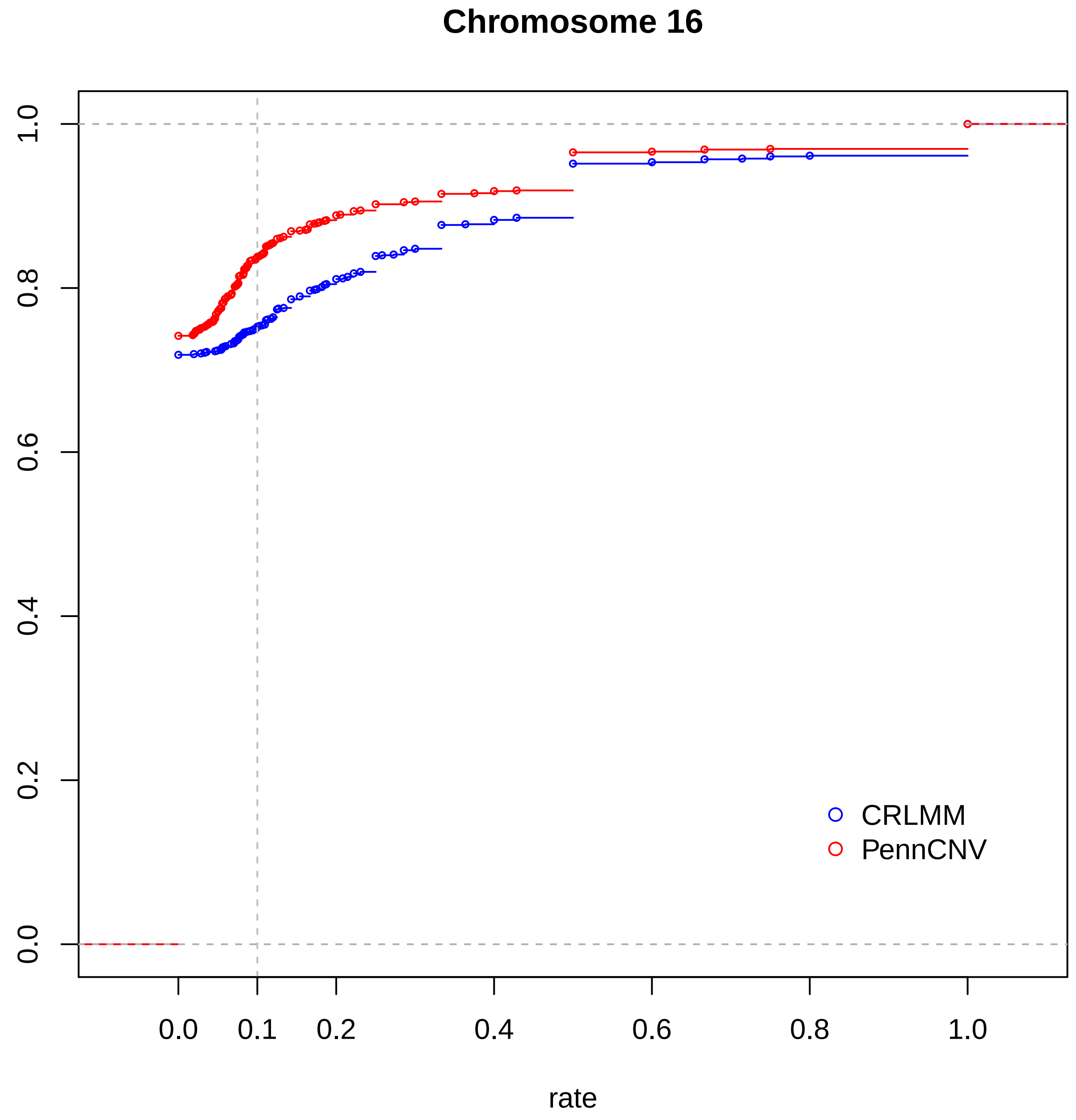
Chromosome 16
0.0
0.2
0.4
0.6
0.8
1.0
0.0
0.1
0.2
0.4
0.6
0.8
1.0
rate
CRLMM
PennCNV

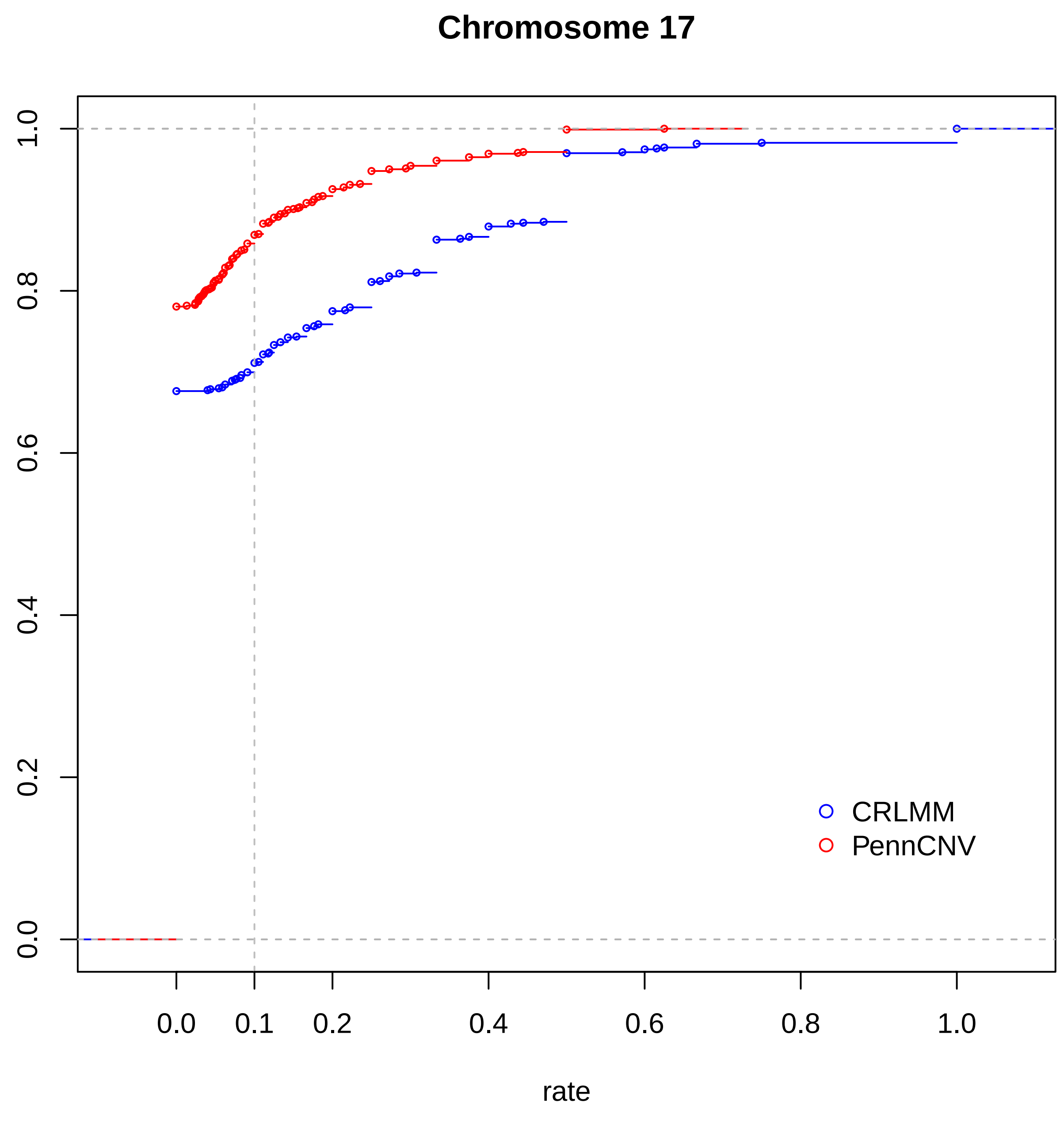
Chromosome 17
1.0
0.8
0.6
0.4
0.2
0.0
0.0
0.1
0.2
0.4
0.6
0.8
1.0
rate
CRLMM
PennCNV

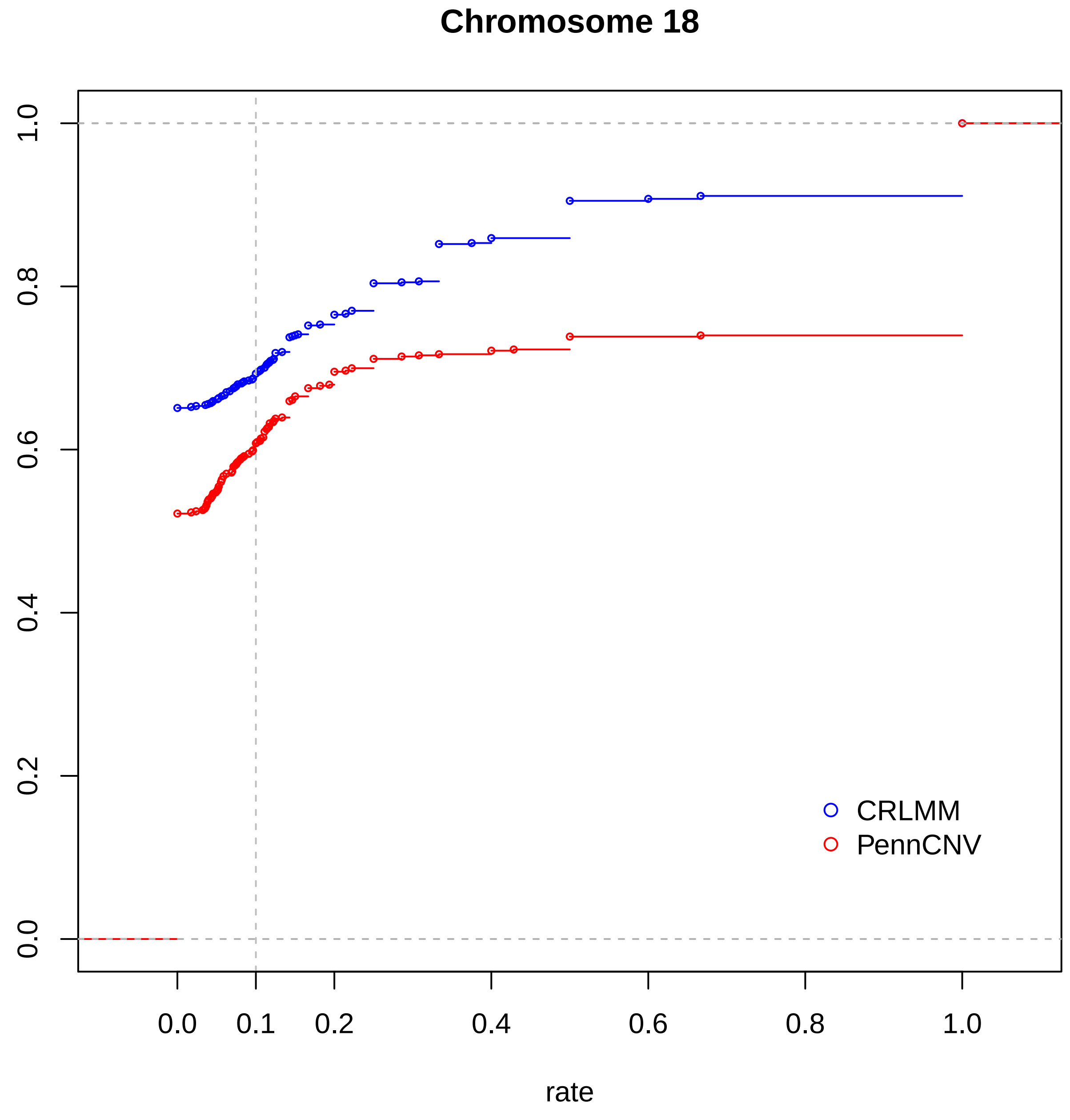

Chromosome 18
0.0
0.2
0.4
0.6
0.8
1.0
0.0
0.1
0.2
0.4
0.6
0.8
1.0
rate
CRLMM
PennCNV

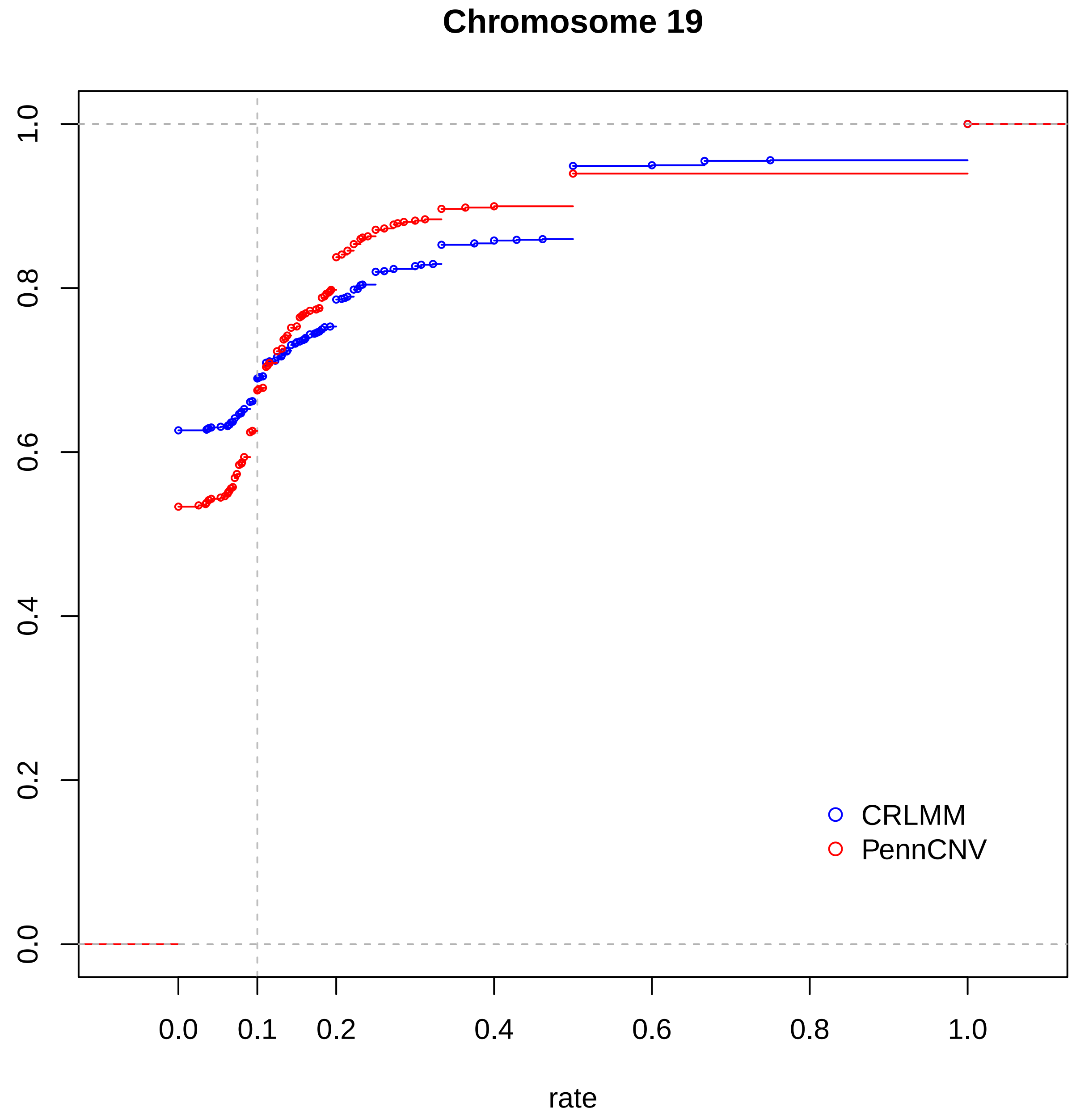
Chromosome 19
0.0
0.2
0.4
0.6
0.8
1.0
0.0
0.1
0.2
0.4
0.6
0.8
1.0
rate
CRLMM
PennCNV

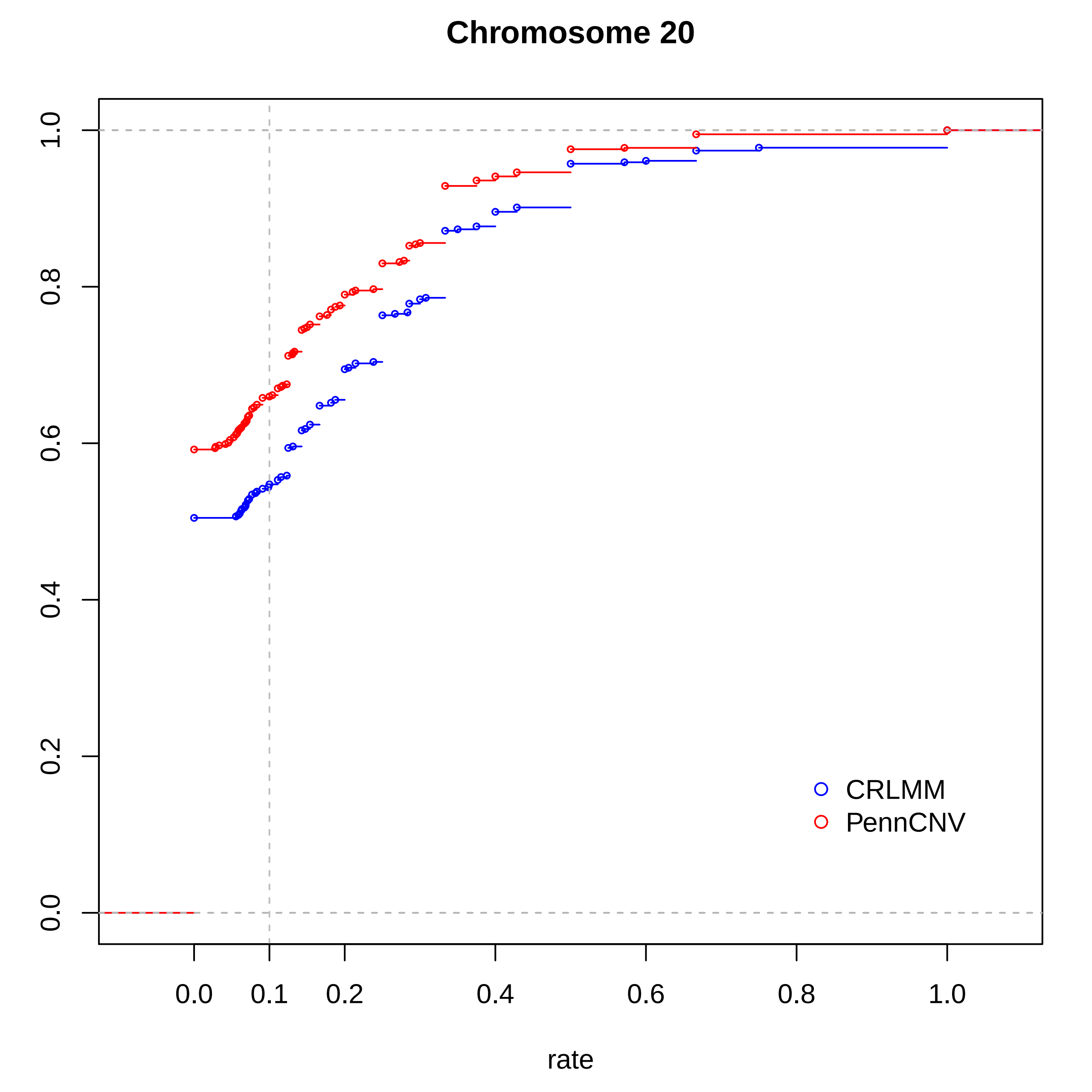

Chromosome 20
1.0
0.8
0.6
0.4
0.2
0.0
0.0
0.1
0.2
0.4
0.6
0.8
1.0
rate
CRLMM
PennCNV

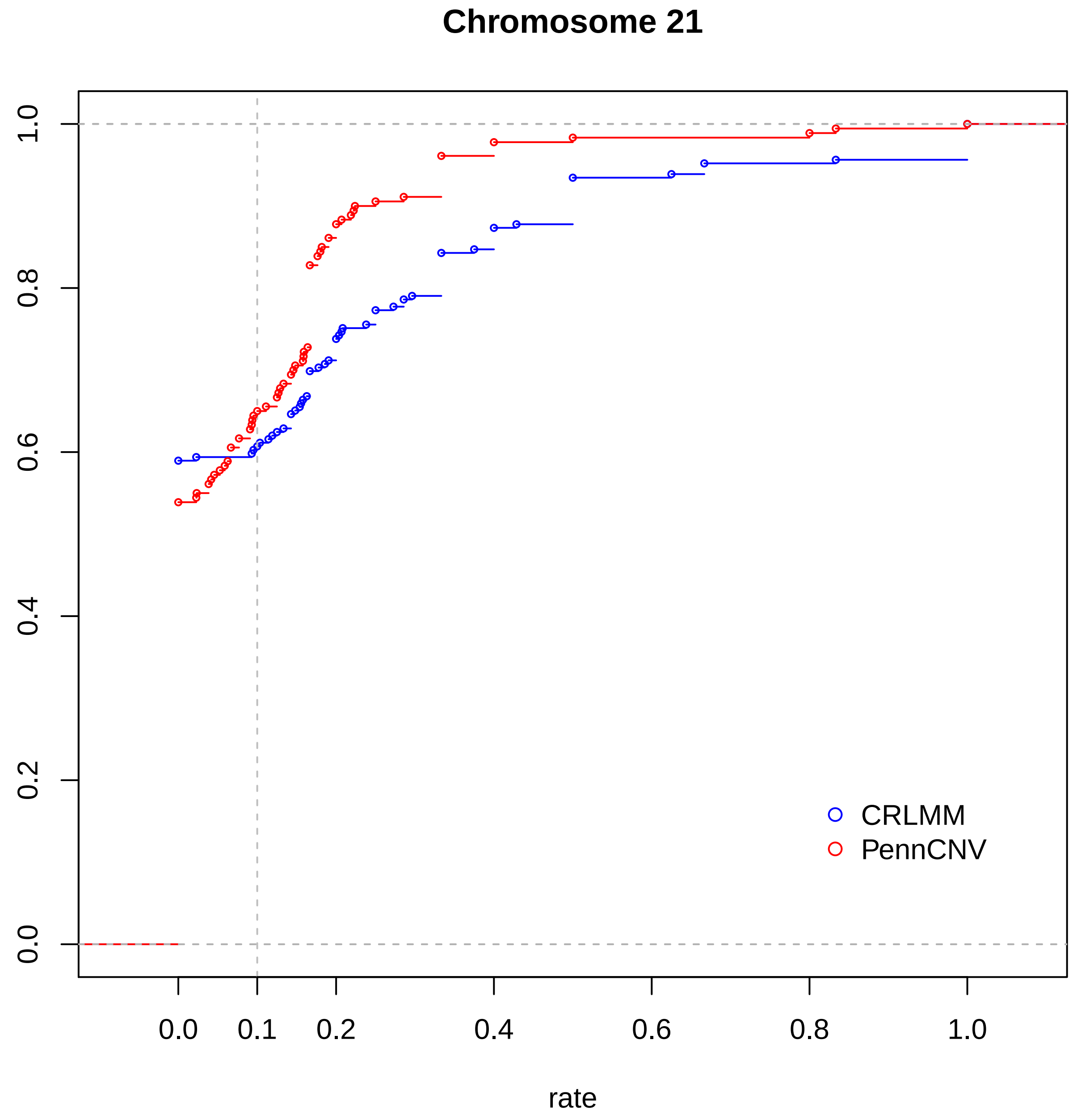

Chromosome 21
1.0
0.8
0.6
0.4
0.2
0.0
0.0
0.1
0.2
0.4
0.6
0.8
1.0
rate
CRLMM
PennCNV

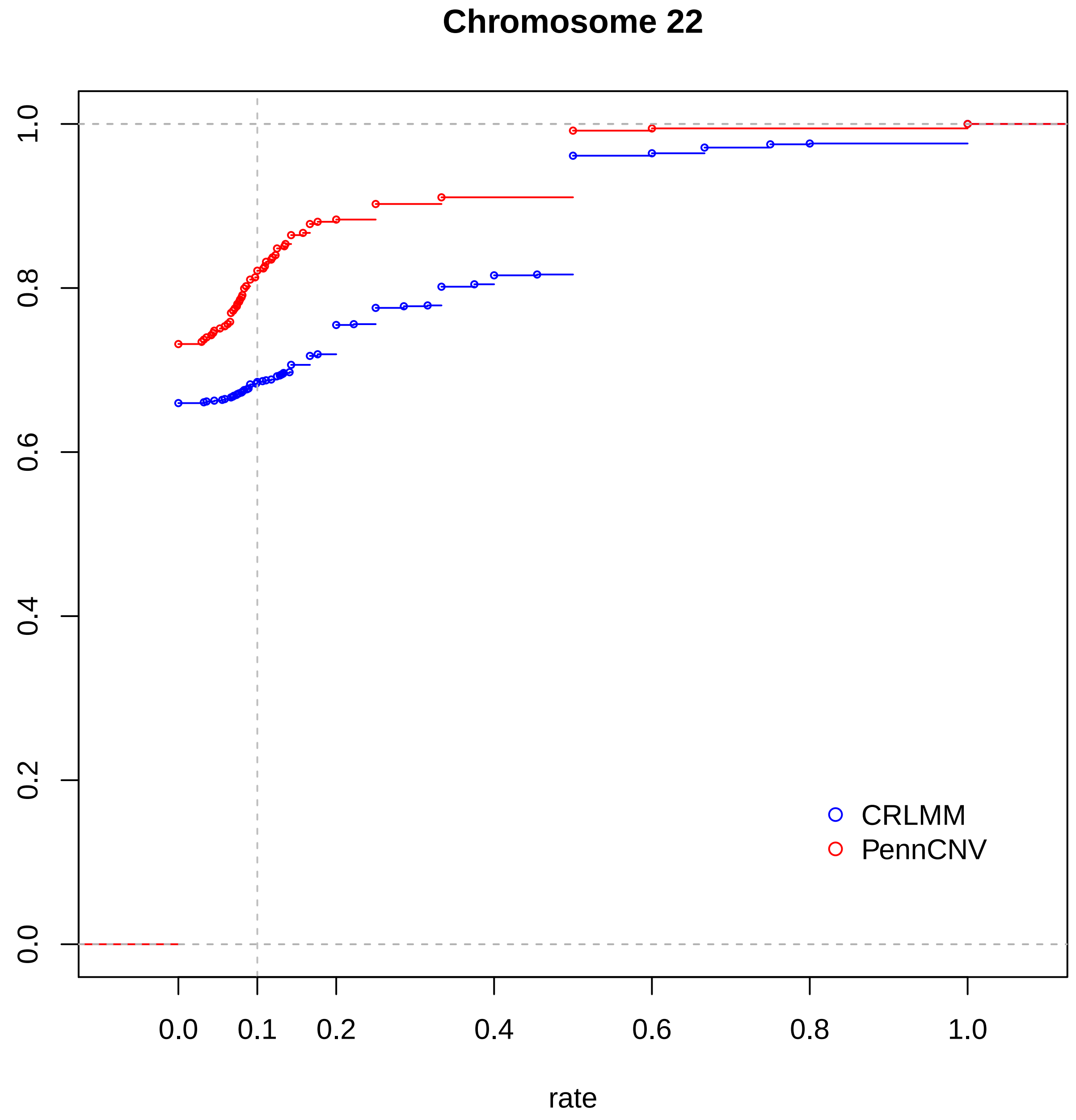

Chromosome 22
0.0
0.2
0.4
0.6
0.8
1.0
0.0
0.1
0.2
0.4
0.6
0.8
1.0
rate
CRLMM
PennCNV
