# Supplementary material for: Software comparison for evaluating genomic copy number variation for Affymetrix 6.0 SNP array platform
Source: BMC Bioinformatics. 2011 May 31;12:220. doi: 10.1186/1471-2105-12-220 (PMC3146450; doi:10.1186/1471-2105-12-220)
Supplement: Additional file 2 — Software settings for Aroma.Affymetrix, Affymetrix Power Tools (APT), PennCNV and CRLMM/VanillaIce. [file 1471-2105-12-220-S2.PDF]

Aroma.Affymetrix

```
## Move to local space (space where the data is)
setwd('/data1/bsi/hypertension/s104892.genoa/eval_cnv/aroma.affymetrix')

## Load all libraries required by aroma.affymetrix
library(rlocal)
library(R.methodsS3
library(sfit
library(R.oo
library(R.utils)
library(R.cache)
library(digest)
library(R.filesets)
library(R.rsp)
library(matrixStats)
library(aroma.light)
library(aroma.core)
library(R.huge)
library(aroma.apd)
library(aroma.affymetrix)

## Notes:
## Results cached to file are stored by default in the so called 'cache root path': ~/.Rcache
## This path can be modified by saying setCacheRootPath('/tmp/.Rcache/')
cache.dir <- '/tmp/m047314/.Rcache/'; mkdir(cache.dir)
setCacheRootPath(cache.dir)

options(digits=4)
## log <- verbose <- Arguments$getVerbose(-8, timestamp=TRUE)
verbose <- Arguments$getVerbose(-8, timestamp=TRUE)
cdf <- AffymetrixCdfFile$byChipType("GenomeWideSNP_6", tags="Full")
csR <- AffymetrixCelSet$byName(name="run1", cdf=cdf)


## Subset array to test
## csR <- extract(csR, ) ## value of  is changed by script "run.CRMAv2.sh"
## print(csR)

##  From here, proceed with CRMA v2 as usual.  It is extremely important
##  that you use the target="zero" arguments throughout, otherwise it will
##  not be a truly single-array method.

## #######
## Step 1
## #######
cat('\n## Step 1 - Calibration for crosstalk between allele probe pairs\n')
acc <- AllelicCrosstalkCalibration(csR, model="CRMAv2")
csC <- process(acc, verbose=verbose)
```

```
## #######
## Step 2
## #######
## It is extremely important that you use the target="zero" arguments throughout,
## otherwise it will not be a truly single-array method.
cat('\n## Step 2 - Normalization for nucleotide-position probe sequence effects\n')
bpn <- BasePositionNormalization(csC, target="zero")
csN <- process(bpn, verbose=verbose)


## #######
## Step 3
## #######
cat('\n## Step 3 - Probe summarization\n')
plm <- AvgCnPlm(csN, mergeStrands=TRUE, combineAlleles=TRUE)
if (length(findUnitsTodo(plm)) > 0) {
  units <- fitCnProbes(plm, verbose=verbose);
  units <- fit(plm, verbose=verbose, ram=400) ## value of ram=400 is change by script
"run.CRMAv2.sh"
}

cat('\n#getChipEffectSet\n')
ces <- getChipEffectSet(plm)

## #######
## Step 4
## #######
cat('\n## Step 4 - Normalization for PCR fragment-length effects\n')
fln <- FragmentLengthNormalization(ces, target="zero")
cesN <- process(fln, verbose=verbose)
```

Affymetrix Power Tools (APT)

```
##############################################################################
###
## Run Affy Power Tool to obtain the copy number values
##############################################################################
###
## use N=270 Hapmap samples as the reference population

/usr/local/biotools/apt/current/bin/apt-copynumber-workflow \
    --adapter-type-normalization true \
    --reference-input $path/lib/GenomeWideSNP_6.hapmap270.na29.r1.a5.ref \
    --cdf-file $path/lib/GenomeWideSNP_6.cdf \
    --chrX-probes $path/lib/GenomeWideSNP_6.chrXprobes \
    --chrY-probes $path/lib/GenomeWideSNP_6.chrYprobes \
    --special-snps $path/lib/GenomeWideSNP_6.specialSNPs \
```

```
    --netaffx-snp-annotation-file $path/lib/GenomeWideSNP_6.na29.annot.csv \
    --netaffx-cn-annotation-file $path/lib/GenomeWideSNP_6.cn.na29.annot.csv \
    --delete-files true \
    --out-dir $path/results/roch/copynumber-workflow-results \
    --text-output true \
    --cnchp-output true \
    --cel-files $listfile
```

PennCNV

```
####################################################################################
###
## Run Affy Power Tool to obtain the contrast QC values
## - this will tell you which samples aren't considered 'good' for
##   the next step, genotype calling with the birdseed algorithm
####################################################################################
###


/usr/local/biotools/apt/current/bin/apt-geno-qc \
 --cdf-file $path/lib/GenomeWideSNP_6.cdf \
 --qcc-file $path/lib/GenomeWideSNP_6.r2.qcc \
 --qca-file $path/lib/GenomeWideSNP_6.r2.qca \
 --chrX-probes $path/lib/GenomeWideSNP_6.chrXprobes \
 --chrY-probes $path/lib/GenomeWideSNP_6.chrYprobes \
 -o $outpath/geno-qc-out.txt \
 --cel-files $listfile

####################################################################################
###
## Run Affy Power Tool to run birdseed and the newer CN based gender caller:
####################################################################################
###

/usr/local/biotools/apt/current/bin/apt-probeset-genotype \
 -c $path/lib/GenomeWideSNP_6.cdf \
 -a birdseed-v2 \
 --read-models-birdseed $path/lib/GenomeWideSNP_6.birdseed-v2.models \
 --special-snps $path/lib/GenomeWideSNP_6.specialSNPs \
 --chrX-probes $path/lib/GenomeWideSNP_6.chrXprobes \
 --chrY-probes $path/lib/GenomeWideSNP_6.chrYprobes \
 --set-gender-method cn-probe-chrXY-ratio \
 -o $outpath \
 --use-disk=false \
 --block-size 70000 \
 --verbose 2 \
 --cel-files $listfile
```

```
############################################################################
###
## Run Affy Power Tool to extract the allele-specific signal values:
############################################################################
###
/usr/local/biotools/apt/current/bin/apt-probeset-summarize \
 --cdf-file $path/lib/GenomeWideSNP_6.cdf \
 --analysis quant-norm.sketch=50000, pm-only,med-polish,expr.genotype=true \
 --target-sketch $path/lib/hapmap.quant-norm.normalization-target.txt \
 --out-dir $outdir \
 --cel-files $celfiles

########################################################################
# run the penncnv-affy script to generate canonical genotype clusters
########################################################################

# Create sex_file used by generate_affy_geno_cluster.pl
sexfile=$tmpDir/sex_file

generate_affy_geno_cluster.pl $calls $conf $quant -locfile $locfile -sexfile $sexfile -out $clust

#############################################################
# run the penncnv-affy script to normalize genotype clusters
#############################################################

llrbaf=$results/gw6.llr_baf.txt
normalize_affy_geno_cluster.pl $clust $quant -locfile $locfile -out $llrbaf

####################################################################
# run the penncnv-affy script kcolumn to split gw6.llr_baf.txt file
#######################################@############################

kcol=$tmpDir/kcol
if [ ! -e $kcol ]; then mkdir -p $kcol; fi
kcolumn.pl $llrbaf split 2 -tab -head 3 -name -out $kcol/gw6

#####################
# Generate CNV calls
#####################

signallist=$tmpDir/signallistfile
ls -1 $kcol | grep gw6 | sed "s|^|$kcol/|g" > $signallist

# shorten script
hmm=$pennRoot/lib/affygw6.hmm
rawcnv=$results/gw6.rawcnv
log=$results/gw6.log
# run Perl
detect_cnv.pl -test -hmm $hmm -pfb $locfile -list $signallist -log $log -out $rawcnv
```

CRLMM

```
mk.crlmm.batch <- function(bat=0, CHR=22, logR=T, hmm=F){

  ## Load packages
  ## The following packages are required:
  library(Biobase)
  library(crlmm) ## 1.4.3
  library(genomewidesnp6Crlmm)

  outdir <-
paste('/data1/bsi/hypertension/s104892.genoa/eval_cnv/crlmm/results/roch/batch',bat,sep='')
  load(paste(outdir,'/crlmmSetList_',CHR,'.rda',sep=''))

  ## Locus- and allele-specific estimates of copy number. Load the object for chromosome 22
and compute copy number:
  ## CHR <- 22
  if (!exists("crlmmSetList")) load(file.path(outdir, paste("crlmmSetList_", CHR, ".rda", sep = "")))
  show(crlmmSetList)

  if (length(crlmmSetList) == 2) {
    crlmmSetList <- update(crlmmSetList, CHR = CHR)
  }
  show(crlmmSetList)


  if(logR){
    cat('computing log R ratios...\n')

    ## Total copy number at both polymorphic and nonpolymorphic loci:
    cn <- copyNumber(crlmmSetList)

    ## ######################################################
    ## Here starts the actual making of crlmm RData object
    ## ######################################################

    crlmm.cn <- data.frame(cn)

    ## Rename pins (shorter)
    pins <- get.pinPlate(sampleNames(crlmmSetList), return=T)$pin
    names(crlmm.cn) <- pins

    ## Add columns
    crlmm.cn$pos <- position(crlmmSetList)
    crlmm.cn$chr <- chromosome(crlmmSetList)
    crlmm.cn$affyid <- rownames(crlmm.cn)

    ## Remove NAs
    crlmm.cn <- na.omit(crlmm.cn)
```

```
    ## Get Plates
    plates <- get.pinPlate(sampleNames(crlmmSetList), return=T)$plate
    num.plates <- as.numeric(substring(plates,2,4))

    ## Create an object with pins, plates and num.plates
    crlmm.pl8s <- data.frame(cbind(pins, plates))
    crlmm.pl8s$num.plates <- num.plates

    ## Reorder
    crlmm.cn <- crlmm.cn[,c('affyid','pos','chr',pins)]

    return(list(crlmm.cn=crlmm.cn, crlmm.pl8s=crlmm.pl8s))
  }

  if(hmm){
    cat('computing hidden markov model...\n')

    ## ######################
    ## A hidden Markov model
    ## ######################
    library(VanillaICE)
    copyNumberStates <- 0:3

    if (!exists("emission.cn")) {
      emission.cn <- suppressWarnings(crlmm:::computeEmission(crlmmSetList,
                                                        copyNumberStates))
      dim(emission.cn)
    }

    initialP <- rep(1/length(copyNumberStates), length(copyNumberStates))
    tau <- transitionProbability(chromosome = chromosome(crlmmSetList),
                                position = position(crlmmSetList), TAUP = 1e+08)

    ## The viterbi algorithm is used to identify the sequence of states that maximizes
    ## the likelihood:
    if (!exists("hmmPredictions")) {
      hmmPredictions <- viterbi(emission = emission.cn,
                              initialStateProbs = log(initialP), tau = tau[, "transitionPr"], arm = tau[, "arm"],
                              normalIndex = 3, normal2altered = 0.01,
                              altered2normal = 1, altered2altered = 0.001)
    }

    brks <- breaks(x=hmmPredictions,
                        states=copyNumberStates, position=tau[, "position"],
                        chromosome=tau[, "chromosome"])

    return(list(brks=brks, hmm=hmmPredictions))
  }
}
```
